# Reference genome and comparative genome analysis for the WHO reference strain for *Mycobacterium bovis* BCG Danish, the present tuberculosis vaccine

**DOI:** 10.1186/s12864-019-5909-5

**Published:** 2019-07-08

**Authors:** Katlyn Borgers, Jheng-Yang Ou, Po-Xing Zheng, Petra Tiels, Annelies Van Hecke, Evelyn Plets, Gitte Michielsen, Nele Festjens, Nico Callewaert, Yao-Cheng Lin

**Affiliations:** 1 VIB-UGhent Center for Medical Biotechnology, Technologiepark-Zwijnaarde 71, 9052 Ghent, Belgium; 2 Department of Biochemistry and Microbiology, Ghent University; Technologiepark-Zwijnaarde 71, 9052 Ghent, Belgium; 3 Biotechnology Center in Southern Taiwan, Academia Sinica, Tainan, 74145 Taiwan; 4 Agricultural Biotechnology Research Center, Academia Sinica, Tainan, 74145 Taiwan

**Keywords:** Tuberculosis, Live vaccines, BCG, Next-generation sequencing, Complete genomic sequence, Genetic differences, Tandem duplications

## Abstract

**Background:**

*Mycobacterium bovis* bacillus Calmette-Guérin (*M. bovis* BCG) is the only vaccine available against tuberculosis (TB). In an effort to standardize the vaccine production, three substrains, i.e. BCG Danish 1331, Tokyo 172–1 and Russia BCG-1 were established as the WHO reference strains. Both for BCG Tokyo 172–1 as Russia BCG-1, reference genomes exist, not for BCG Danish. In this study, we set out to determine the completely assembled genome sequence for BCG Danish and to establish a workflow for genome characterization of engineering-derived vaccine candidate strains.

**Results:**

By combining second (Illumina) and third (PacBio) generation sequencing in an integrated genome analysis workflow for BCG, we could construct the completely assembled genome sequence of BCG Danish 1331 (07/270) (and an engineered derivative that is studied as an improved vaccine candidate, a SapM KO), including the resolution of the analytically challenging long duplication regions. We report the presence of a DU1-like duplication in BCG Danish 1331, while this tandem duplication was previously thought to be exclusively restricted to BCG Pasteur. Furthermore, comparative genome analyses of publicly available data for BCG substrains showed the absence of a DU1 in certain BCG Pasteur substrains and the presence of a DU1-like duplication in some BCG China substrains. By integrating publicly available data, we provide an update to the genome features of the commonly used BCG strains.

**Conclusions:**

We demonstrate how this analysis workflow enables the resolution of genome duplications and of the genome of engineered derivatives of the BCG Danish vaccine strain. The BCG Danish WHO reference genome will serve as a reference for future engineered strains and the established workflow can be used to enhance BCG vaccine standardization.

**Electronic supplementary material:**

The online version of this article (10.1186/s12864-019-5909-5) contains supplementary material, which is available to authorized users.

## Background

The BCG live attenuated TB vaccine is one of the oldest and most widely used vaccines in human medicine. Each year, BCG vaccines are administered to over 100 million newborns (i.e. 75% of all newborns on the planet). The original BCG strain was developed at the Pasteur Institute, through attenuation of the bovine TB pathogen *M. bovis,* by 231 serial passages on potato slices soaked in glycerol-ox bile over a time-span of 13 years [1]. After its release for use in 1921, this BCG Pasteur strain was distributed to laboratories around the world and different laboratories maintained their own daughter strains by passaging. Over the years, different substrains arose with different protective efficacy [2, 3]. The establishment of a frozen seed-lot system in 1956 and the WHO (World Health Organization) recommendation of 1966 that vaccines should not be prepared from cultures that had undergone > 12 passages starting from a defined freeze-dried seed lot, halted the accumulation of additional genetic changes [1]. In an effort to further standardize the vaccine production and to prevent severe adverse reactions related to BCG vaccination, three substrains, i.e. BCG Danish 1331, Tokyo 172–1 and Russia BCG-1 were established as the WHO reference strains in 2009 and 2010 [4]. Of these, the BCG Danish 1331 strain is the most frequently used one, and it also serves as a basis of most current 'next-generation' engineering efforts to improve the BCG vaccine or to use it as a 'carrier' for antigens of other pathogens [5, 6].

Complete genome elucidation of BCG strains is challenging by the occurrence of large genome segment duplications and a high GC content (65%). Therefore, no fully assembled reference genome is yet available for BCG Danish, only incomplete ones [7, 8], which hinders further standardization efforts. In this study, we set out to determine the completely assembled genome sequence for BCG Danish and meanwhile, to establish a current-generation sequencing-based workflow to analyze genomes of BCG Danish-derived engineered strains.

## Results

### General genomic features of the whole genome sequence for BCG Danish 1331 (07/270)

**Fig. 1:**
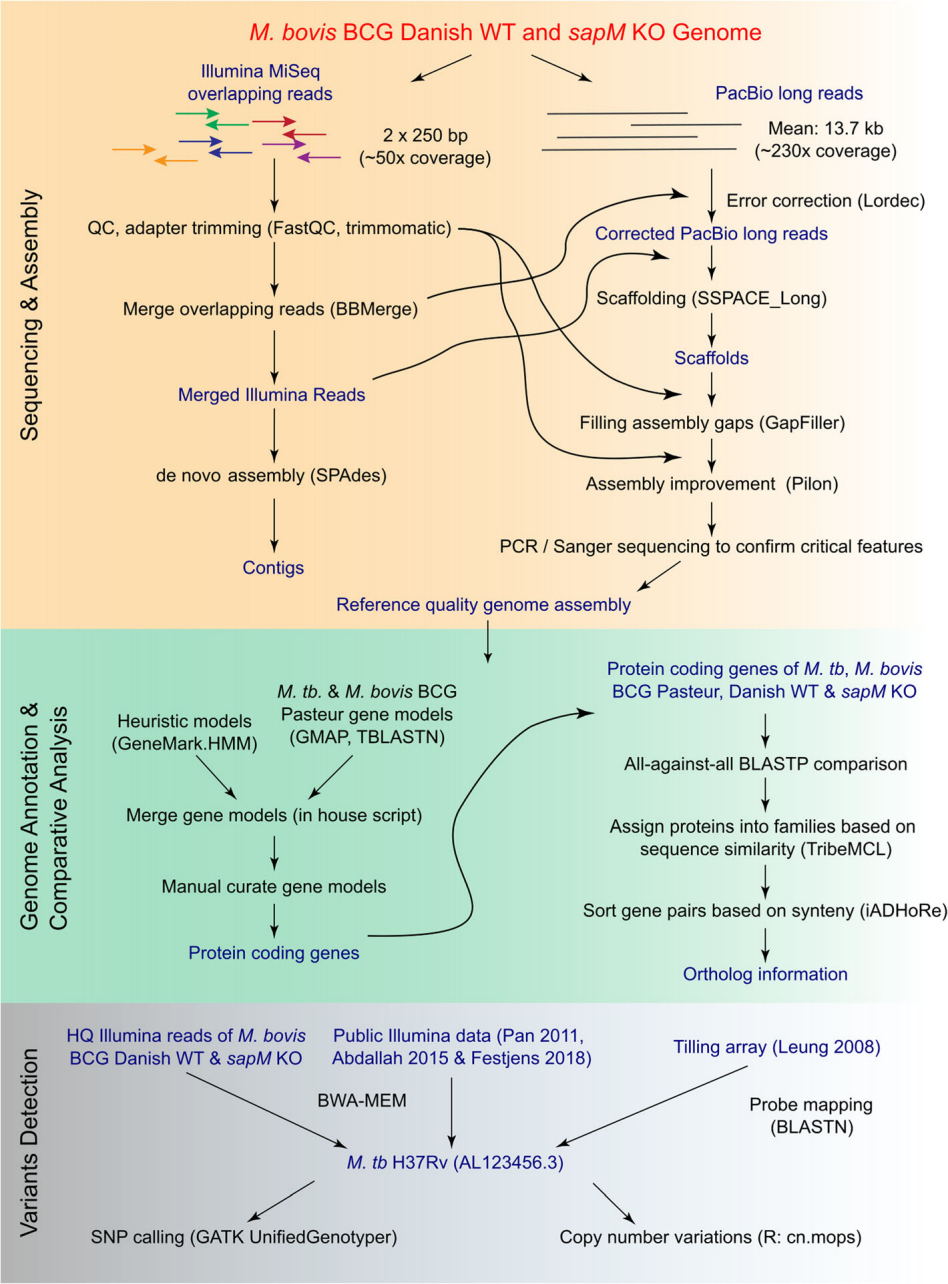
Genome analysis pipeline

**Fig. 2:**
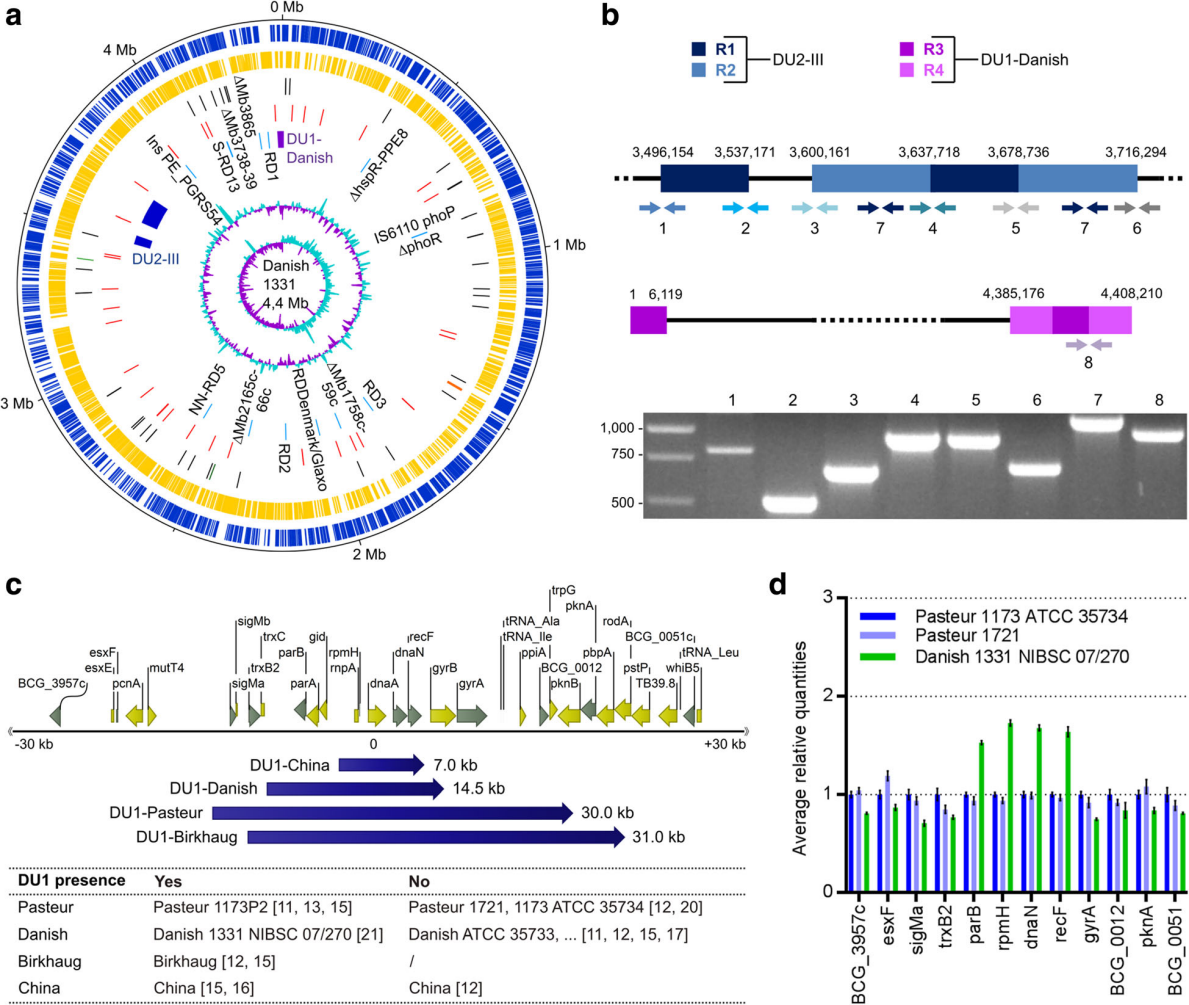
Organization of the BCG Danish 1331 (07/270) genome, focusing on the DU1 and DU2. **a** Circular representation of the BCG Danish chromosome. The scale is shown in megabases on the outer black circle. Moving inward, the next two circles show forward (dark blue) and reverse (yellow) strand CDS (coding sequence). The next circle shows 3 rRNAs (5S, 16S and 23S; orange), 45 tRNAs (black), 1 tmRNA (ssrA; green) and 1 ncRNA (rnpB; dark green3), followed by 42 SNPs (red) detected between BCG Danish and Pasteur. The subsequent circle shows DU2-III (dark blue), DU1-Danish (purple) and RD (light blue, names of RD in black) that are typical for BCG Danish. The two inner circles represent G + C content and GC skew. **b** Organization of the two tandem duplications in BCG Danish and confirmation by PCR. The DU2 is made up by two repeats (R1 and R2), as well as the DU1-Danish (R3 and R4). Used primer pairs (1–8) to validate their organization are indicated. **c** Visual representation of the *oriC* with position and size of DU1-China, −Danish, −Pasteur and -Birkhaug. The table indicates which substrains have the DU1. **d** Copy-number analysis of genes (indicated in grey in subfigure c) in and surrounding the DU1 region for Pasteur 1173 ATCC 35734, Pasteur 1721 and Danish 1331 NIBSC 07/270. The represented data are averages (± SD) of four technical replicates

The BCG Danish 1331 (07/270) strain genome sequence was assembled by combining second (Illumina) and third (PacBio) generation sequencing technologies in an integrated bioinformatics workflow **(**Fig. 1, see Methods). Ambiguous regions were locally reassembled and/or experimentally verified (Additional file 1: Table S1). In all cases, the experimental validation confirmed the assembly, demonstrating that this integration of sequencing data types and bioinformatics workflow is adequate for high-GC mycobacterial genomes. The single circular chromosome is 4,411,814 bp in length and encodes 4084 genes, including 4004 genes encoding for proteins, 3 genes for rRNA (5S, 16S and 23S), 45 genes for tRNA, 1 tmRNA gene (ssrA), 1 ncRNA gene (rnpB) and 30 pseudogenes (Fig. 2a). Compared to the reference genome sequence of BCG Pasteur 1173P2, 42 SNPs were identified, including 24 non-synonymous SNPs, 9 synonymous SNPs and 9 SNPs in the intergenic region (Additional file 1: Table S2). For all the genes containing missense and/or nonsense SNPs, we attempted to validate the SNPs via PCR and Sanger sequencing (26 SNPs affecting 19 genes) (Additional file 1: Table S3). In all cases where the validation experiment yielded interpretable quality results (i.e. not hindered by highly repetitive and/or highly GC-rich regions), these mutations were all validated (15 SNPs affecting 15 genes), demonstrating that the generated genome has very high per-base accuracy. Genetic features determinative for the BCG Danish substrain, as described by Abdallah et al. [8], were identified, including the region of difference (RD) Denmark/Glaxo and the DU2 type III, that was completely resolved in the assembly (Fig. 2a-b). Additionally, a 1 bp deletion in Mb3865 and a 465 bp insertion in PE_PGRS54 compared to BCG Pasteur were found. The organization of 2 repeats (A and B) in PE_PGRS54 has been reported to differ between the BCG strains [9]. We report a A-A-B-B-B-B organization for BCG Danish in contrast to BCG Tokyo (A-A-B-B-B) and BCG Pasteur (A-B-B-B-B). Previously, two separate genetic populations for BCG Danish 1331 have been described, which differ in the SenX3-RegX3 region (having 2 or 3 repeats of 77 bp) [10]. For BCG Danish 1331 07/270, we documented only 3 repeats of 77 bp (Additional file 1: Figure S1). Two features described by Abdallah et al. [8] to be determinative for BCG Danish were not identified, namely the rearrangement of the *fadD26-pssA* gene region and a 894 bp deletion in Mb0096c-Mb0098c. In addition, a 399 bp instead of a 118 bp insertion was detected in *leuA*, giving 12 direct repeats of 57 bp, as in the Pasteur strain (previously denoted as S-RD13 [11]). These three regions were characterized by the presence of inherent repeat structures. Furthermore, these genome regions contained assembly gaps in the assembly for BCG Danish published with the study of Abdallah et al. [8, 12], so it is likely that our long-read based genome is more accurate in these challenging regions.

### The DU1 in BCG strains

**Fig. 3:**
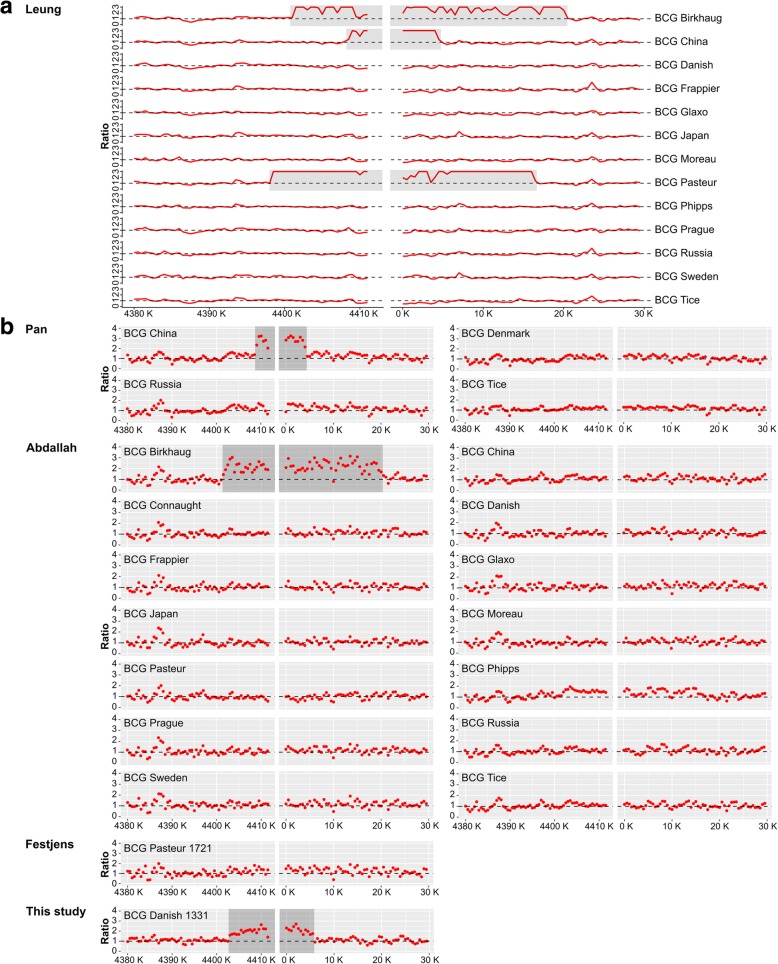
DU1 duplication detection in BCG strains. Tiling array data (**a**) from Leung et al. 2008 [15] and Illumina sequencing data (**b**) for BCG Danish 1331 (this study) as well as published genome data from Pan et al. 2011 [16–19], Abdallah et al. 2015 [12] and Festjens et al. 2019 [20] were reanalyzed for the presence of a DU1 in the region of the *oriC*. These references were chosen as they contain BCG Danish or BCG Pasteur genome sequencing data. The graphs in (**a**) depict the ratio of the reference (*M. tb* H37Rv) probe intensity (Cy5) divided by the test (BCG strain) probe intensity as originally presented in Leung et al. 2008 [14]. The graphs in (**b**) depict the ratio of mean whole genome read coverage divided by the mean read coverage in 500 bp window size. Detection of a DU1-like duplication in BCG Pasteur 1173P2 [15], Birkhaug [12, 15], Danish 1331 07/270 (this study) [21] and BCG China [15, 16] sequencing data, indicated in grey. No detection of DU1-duplication for other BCG Pasteur [12, 20], Danish [12, 17] and China [12] sequencing data

Two large tandem chromosomal duplications characterize the BCG strains; the DU2 and DU1. While four different forms of the DU2 exist, the DU1 is supposed to be exclusively present in BCG Pasteur [11, 13, 14]; it spans the chromosomal origin of replication or *oriC* (*dnaA-dnaN* region) and encodes key components of the replication initiation and cell division machinery. Surprisingly, we detected a DU1-like duplication of 14,577 bp in BCG Danish (Fig. 2). This finding was validated by performing a copy-number analysis of genes in and surrounding the DU1-like duplication (Fig. 2d). To adapt an unambiguous terminology, we considered all duplications spanning the *oriC* as DU1, while specifying the strain in which the duplication was found. Investigation of other publicly available data for BCG Danish did not show presence of a DU1 (Figs. 2c and 3), indicating that only the Danish 1331 substrain deposited as the WHO reference at the National Institute for Biological Standards and Control (NIBSC) contains this duplication. Additional inconsistencies in DU1 presence/absence were detected by reanalyzing publicly available data [12, 15–20] (Figs. 2c and 3): in contrast to what is concluded in the literature, we found that the public data show that there are BCG Pasteur substrains with a DU1 (data [15]) and others without a DU1 (data [12, 20]). Similarly, experimental analysis of our in-house Pasteur strains (1721, 1173 ATCC 35734) showed absence of a DU1 (Fig. 2d). Additionally, a DU1-China was detected in some data sources [15, 16], but not in others [12], which is likely explained by the use of two different substrains of BCG that are both named BCG China [8]. DU1-Birkhaug was consistently detected in all reported sequencing data of that BCG strain.

### Characterization of a derivative of BCG Danish 1331, the *sapM* KO

**Fig. 4:**
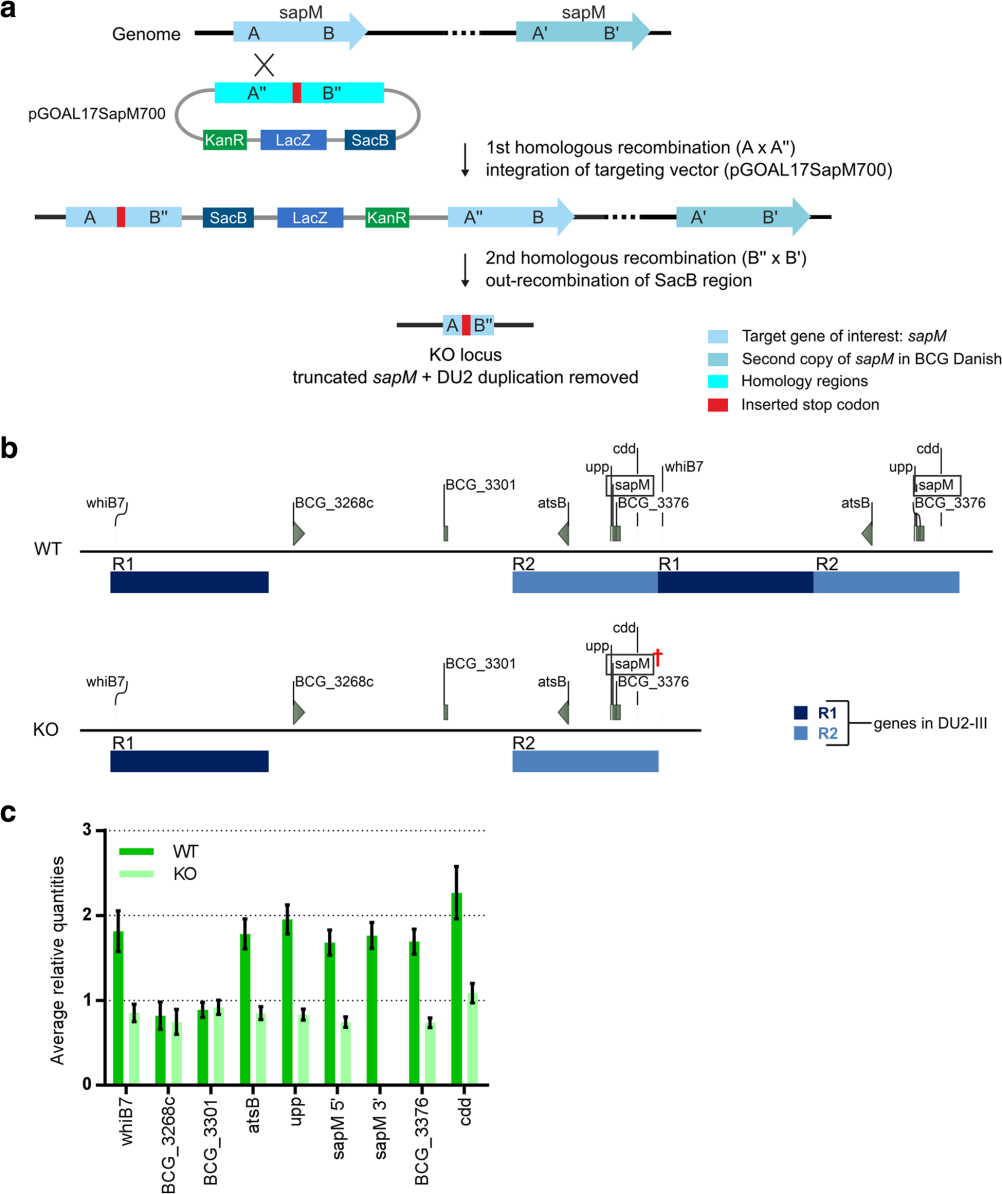
BCG Danish 1331 *sapM* KO has lost the DU2 to form the *sapM* KO locus. **a** Illustration of the outrecombination of the DU2 duplicated genomic region in the formation of the BCG Danish 1331 *sapM* KO from BCG Danish 1331 WT, containing two *sapM* loci, due to the presence of the *sapM* locus in the DU2*.* **b** Genomic organization of the *sapM* region for BCG Danish WT and *sapM* KO. The organization of the DU2 is indicated. †: truncated *sapM*. **c** Copy-number analysis of selected genes (indicated in grey in subfigure **b**) in and surrounding the DU2 via qPCR on gDNA for BCG Danish 1331 WT and *sapM* KO. The represented data are averages (± SD) of four technical replicates

Using the same genome analysis methodology, we determined the complete genome assembly for a KO mutant in the SapM secreted acid phosphatase. Since the *sapM* gene is located in the DU2, the *sapM* locus is present twice in WT cells. The assembly for the *sapM* KO strain did not contain a DU2 repeat, as the KO engineering entirely out-recombined one of the copies of the DU2 to form a single *sapM* KO locus (Fig. 4a). The absence of the DU2 was unequivocally validated by performing a copy-number analysis of multiple genes in and surrounding the DU2 (Fig. 4b). Furthermore, we detected one SNP compared to the parental BCG Danish WT strain, a missense SNP in BCG_3966 or BCGDan_4053 (encoding a conserved hypothetical protein), which was validated by Sanger sequencing (Additional file 1: Table S2 and S3). The single DU2 *sapM* KO is a useful chassis for further vaccine engineering, as another target gene for improving BCG vaccine efficacy (*sigH* ([22]) is novo haploid in this strain, facilitating its future knockout to generate a *sapM*/*sigH* double knockout.

## Discussion

All BCG strains originate from a common ancestor [23], but since then, they have incorporated many gene deletions and evolved gene amplifications (DU1 and DU2), that differentiate the different BCG strains from each other. Several studies on BCG vaccine strains have mapped these genomic changes using a variety of comparative genomic techniques, starting from subtractive genomic hybridization [24] to whole genome sequencing [7, 8, 25], enabling the deciphering of a genealogy of the BCG strains. The study of Abdallah and others used short-read Illumina sequencing data for 14 of the most widely used BCG strains in combination with a large-indel detection pipeline to identify a number of previously unknown deletions and insertions [8]. Most genetic signatures identified for BCG Danish by that study were also found in the complete long read/short read hybrid genome assembly that we generated for BCG Danish 1331. However, some RDs could not be found. We hypothesize that inherent repeat structures in these regions triggered the undue assignment of these regions as RD in the short-read Illumina sequencing dataset. Unequivocal assembly of repeat-containing sequences, clearly requires long sequencing reads, as generated for example by PacBio SMRT sequencing in this study.

In 2001, Bedwell and others identified two substrains admixed in a Copenhagen commercial preparation of the BCG vaccine (a.k.a. BCG Danish 1331) [10]. These two genetic populations differed in the *senX3-regX3* region, having 2 or 3 repeats of 77 bp. We documented only one version for the *senX3-regX3* region, with 3 repeats of 77 bp for the BCG Danish 1331 WHO reference reagent strain. In contrast, Magdalena et al. reported the presence of 2 repeats for a *M. bovis* BCG Danish vaccine strain provided by M. Lagranderie (Institut Pasteur, Paris, France) [26]. These data indicate that different substrains of BCG Danish are in circulation, and that this region probably is genetically drifting. Extensive genomic characterization of the WHO reference reagent for BCG Danish (as provided by this study) will facilitate the identity assurance of the genomic integrity of new lots of the BCG Danish vaccine.

**Fig. 5:**
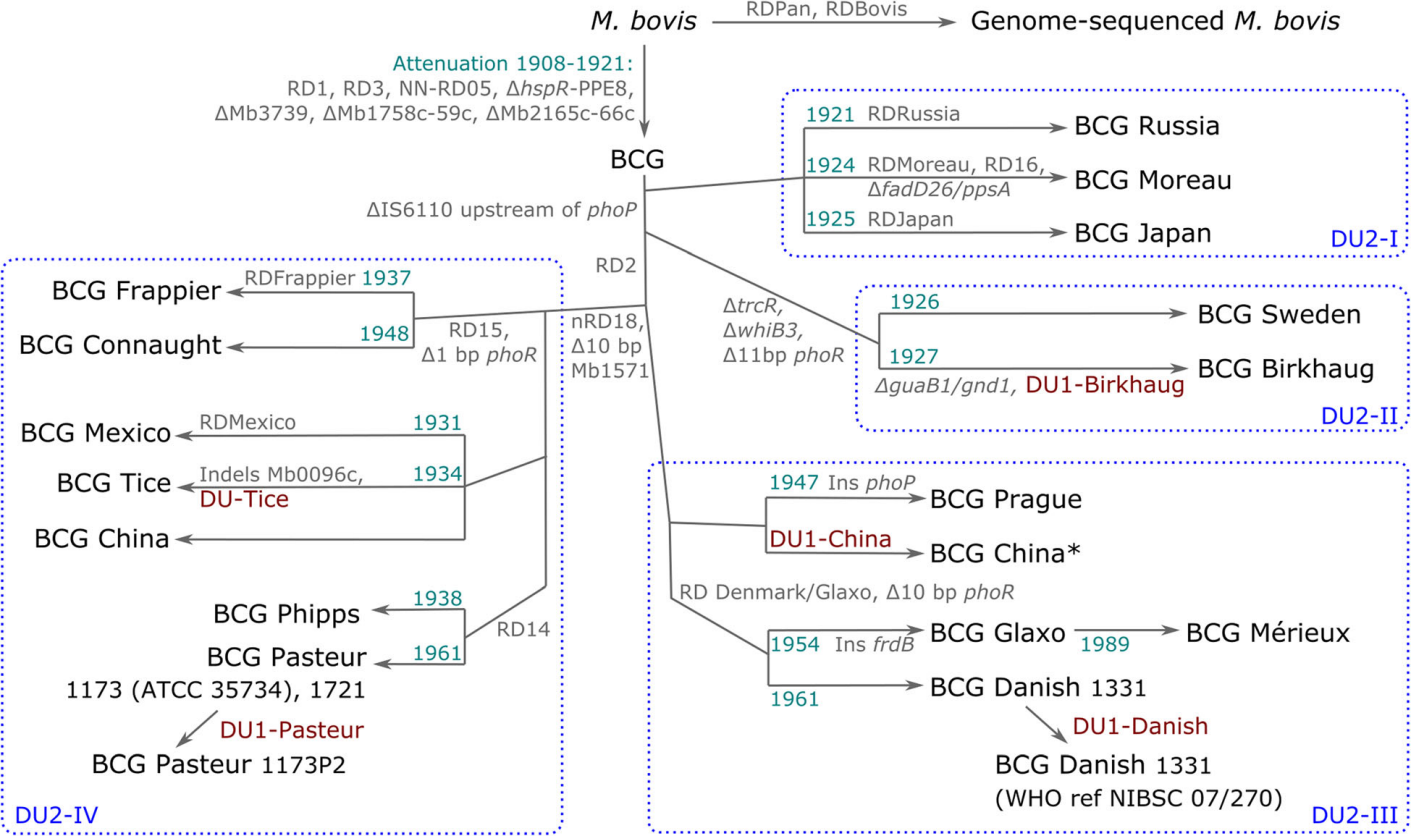
Refined genealogy of BCG vaccine strains**.** The year when the strain was obtained per geographical location is indicated where possible (indigo). The scheme shows regions of difference (RD), insertions (Ins), deletions (‘∆’), indels and tandem duplications (DU), which differentiate the different BCG strains (Additional file 2: Table S8). The blue dashed squares indicate the different DU2-forms, which classify the BCG strains into four major lineages. When the DU1 is not found in all substrains of a certain strain, this is indicated on the scheme. According to the literature, two different substrains of BCG are named BCG China or Beijing [8]. Therefore, the scheme contains two ‘BCG China’ strains: BCG China [8] and BCG China* [7, 14]. Adapted from references [8, 11, 14, 28, 29]. Concerning reference [8], only the RD and deleted genes that could be verified on the assembled genomes [12] are included

Similarly, we document the presence of a DU1-like duplication in this WHO reference BCG strain (DU1-Danish), that has never been reported on before, as the DU1 was thought to be exclusively restricted to BCG Pasteur [11, 23]. Furthermore, we showed that not all BCG Pasteur strains contain the DU1-Pasteur, based on experimental analysis of in-house Pasteur strains and based on reanalysis of publicly available sequencing data. In addition, we detected a DU1-China in one of the two different substrains of BCG that are both named BCG China [8]. Seemingly the *oriC* is prone for duplication, as DU1-like duplications were observed for BCG Pasteur, BCG Birkhaug, BCG China and BCG Danish. The genealogy of BCG strains is thus further complicated by the genomic instability of the *oriC* during in vitro cultivation (Fig. 5, Additional file 2: Table S8). A DU1-like duplication has also been identified in a 'non-vaccine' strain; in a clinical isolate (3281), identified as BCG, a 7-kb region that covered six genes and crossed the *oriC* was repeated three times [27], further indicating that this region is prone to (possibly reversible) duplication. Together, these data underline the importance of the genomic characterization of the BCG vaccine strains, including their dynamic duplications. Furthermore, they demand for the specification of the exact origin of the BCG strain(s) used in studies on this vaccine and the determination of the presence of the RD documented for that strain. The implementation of copy number analysis via qPCR as described here, could allow for easy discrimination whether a certain strain contains a DU1-like duplication or not, instead of requiring next-generation sequencing (more expensive) and bioinformatics analyses (requires expert knowledge).

Until now, no driving factor for the DU1 has been identified, as the DU1 in BCG Pasteur contains 31 genes and none of these genes are expected to give an obvious in vitro growth advantage upon duplication [13]. Perhaps, this could now be elucidated by examining the gene functions of the genes common to all DU1-like duplications. Seven genes are duplicated in all DU1 (DU1-Pasteur, -Birkhaug, -China and -Danish and the DU1-like triplication identified in the clinical isolate BCG 3281), namely BCG_3979c, BCG_3980c, *rnpA*, *rpmH*, *dnaA*, *dnaN* and *recF* (Table 1). It remains however difficult to speculate about the impact of two copies of *oriC* (*dnaA-dnaN* region) on the biology of BCG strains [13]. Bacteria carefully regulate the activity of the initiator protein DnaA and its interactions with the *oriC* to assure correct timing of the chromosome duplication [30]. Therefore, one has assumed that multiple copies of the *oriC* are deleterious, as they can provoke uncoordinated replication [13, 31]. It is known that *M. smegmatis* transformants with two functional *DnaA* gene copies cannot be obtained [31], as observed in both *B. subtilis* [32] and *S. lividans* [33]. However, such an inhibitory effect was not observed when a complete *dnaA* gene was transformed to *M. smegmatis* [34], although Salazar and others questioned whether the construct did not acquire a point mutation or small deletion that inactivated *dnaA* [31]. Until now, no sequence differences were observed between the different copies of the *dnaA-dnaN* region, suggesting that both copies of the origin are functional in vivo. It has been speculated that BCG 3281 (containing 3 copies of the *dnaA-dnaN* region) would likely be capable of enduring greater gene expression burdens in replication [27]. Indeed, as DnaA and *oriC* are so closely genetically linked, duplication of this genomic region is not necessarily the same as just increasing the gene copy number or overexpressing DnaA. It could be envisioned that selection for rapid growth on rich medium may favor or tolerate more rapid genomic replication initiation, but also that this selective advantage may collapse in the face of e.g. nutrient limitation or prolonged stationary phase cultivation. Possibly this is at the heart of the observed unpredictable behavior of this genomic duplication. Confirmation of this hypothesis awaits experimental confirmation.

**Table 1:** Genes (and genome feature) common to all DU1-like duplications (DU1-Pasteur, -Birhaug, -China and -Danish and the DU1-like triplication identified in the clinical isolate BCG 3281)

Gene/feature *M. tb* H37Rv (BCG Pasteur)	Product/Function	Functional category
Rv3921c (BCG3979c)	Probable conserved transmembrane protein	cell wall and cell processes
Rv3922c (BCG3980c)	Possible hemolysin	virulence, detoxification, adaptation
*rnpA*	Ribonuclease P protein component RnpA or RNaseP. RNaseP catalyzes the removal of the 5′-leader sequence from PRE-tRNA to produce the mature 5′ terminus. It can also cleave other RNA substrates such as 4.5S RNA. The protein component plays an auxiliary but essential role in vivo by binding to the 5′-leader sequence and broadening the substrate specificity of the ribozyme.	information pathways
*rpmH*	50S ribosomal protein L34 RpmH. Involved in translation mechanism. This protein is one of the early assembly proteins of the 50S ribosomal subunit.	information pathways
*dnaA*	Chromosomal replication initiator protein DnaA. Plays an important role in the initiation and regulation of chromosomal replication. Binds to the *oriC*; it binds specifically dsDNA at a 9 bp consensus (DnaA box): 5′-TTATC(C/A)A(C/A)A-3′. DnaA binds the *oriC*, ATP and ADP,acidic phospholipids and exhibited weak ATPase activity.	information pathways
*oriC*	Sequence in the genome at which replication is initiated. Contains non-overlapping MtrA- and DnaA-binding boxes. Is located in the *dnaA-dnaN* genomic region [35].	–
*dnaN*	DNA polymerase III (β-chain) DnaN (DNA nucleotidyltransferase). DNA polymerase III is a complex, multichain enzyme responsible for most of the replicative synthesis in bacteria. This DNA polymerase also exhibits 3′ to 5′ exonuclease activity. The β-chain is required for initiation of replication. Once it is clamped onto DNA, it slides freely (bidirectionally and ATP-independently) along duplex DNA.	information pathways
*recF*	DNA replication and repair protein RecF (ssDNA binding protein) is involved in DNA metabolism and recombination; it is required for DNA replication and normal SOS inducibility. Binds preferentially to linear ssDNA. It also seems to bind ATP.	information pathways

Gene information was extracted from Mycobrowser [36]

To demonstrate how the genome analysis methodology, developed in this study, contributes to full characterization of improved BCG-derived engineered vaccines, we applied it to a KO for the SapM secreted acid phosphatase, located in the analytically challenging long duplication region DU2 [11]. Our BCG genome analysis workflow unequivocally demonstrated that the KO engineering had inadvertently out-recombined one of the copies of this DU2 and had furthermore given rise to a single SNP. The out-recombination of the DU2 will most probably not have a dramatic impact on the phenotype of the *sapM* KO, as all the genes are still present as a single copy. One could perhaps expect slower growth of the *sapM* KO in glycerol-containing media, as the DU2 probably arose due to inadvertent selection for increased growth rate on glycerol [11]. *GlpD2*, encoding glycerol-3-phosphate dehydrogenase, is one of the three genes present in all DU2 versions and higher levels of *glpD2* probably gave a growth advantage to strains with duplications [11]. We did not observe a decreased growth rate in the Middlebrook 7H9 standard medium for the *sapM* KO. Perhaps, the growth advantage attributed to the DU2 would only be apparent in Calmette’s glycerol-containing medium, traditionally used to subculture the BCG strains before the introduction of a frozen seed-lot system in 1956 [37]. The effect of the SNP in BCG_3966 (or Rv3909) is hard to estimate. The mutated gene encodes for a conserved hypothetical protein of 802 amino acids and is predicted to be an outer membrane protein [38]. The missense SNP converts the asparagine (located at the end of the protein) in the WT to a threonine in the *sapM* KO (pAsn737Thr). However, as the gene has been found to be essential for in vitro growth of *M. tb* H37Rv [39, 40], we suspect that the protein function is retained. Such unexpected genomic alterations may be more common than thought in engineered live attenuated TB vaccines, but may have so far gone largely unnoticed due to lack of a complete reference genome and/or suitable genome analysis methodology.

The implementation of both short (Illumina) and long (PacBio) sequencing reads in one genome analysis methodology allowed for the straightforward generation of completely assembled genomes of BCG strains. These included the decomposition of the analytically challenging long duplication regions DU1 and DU2, thanks to the inclusion of long sequencing reads, whereas one formerly needed many additional experimentation (Table 2). Furthermore, the generated genome assemblies were highly polished at base level, due to the incorporation of reliable Illumina sequencing reads (single-pass error rate of 0.1%), in addition to the more error-prone PacBio sequencing reads (single-pass error rate of 10–15%) [41, 42]. This methodology is thus currently the most cost-effective strategy that allows to create high-quality BCG genomes, solely based on next-generation sequencing strategies.

**Table 2:** List of *M. bovis* BCG strains for which high per-bp coverage complete genomes are available

Strain	Method	type DU2	DU2 resolved	Accession numbers	Reference	Year of publication
BCG Pasteur 1173P2	**Sanger sequencing (ABI 3700) of a pUC19, two pMAQ1b, a M13 and a cloned shotgun library,** earlier **analysis of BAC clones** had already identified the DU1 and DU2 [13]	IV	yes	PRJEA18059, AM408590	[11]	2007
BCG Tokyo 172	SOLiD (Agencourt Bioscience Corporation) sequencing of **pAGEN vector library** (about 4 kb and 800 bp inserts) and a **fosmid insert library** (about 40 kb)	I	yes (2x)	PRJDA31211, AP010918	[9]	2009
BCG Moreau RDJ	**Sanger (ABI 3730) sequencing of 2 pBluescrip**t **libraries, gap closure via PCR**	I	no	PRJEA70285, AM412059	[43]	2011
BCG Tice ATCC 35743	Illumina genome sequencing,** multiplex PCR and primer walking**	IV	no	PRJNA63839, CP003494	[7]	2011
BCG Mexico 1931	Roche 454 pyrosequencing, **Sanger sequencing of fos-end sequences of 250 fosmids, sequencing of three fosmids and 110 PCR end reads**	IV	yes	PRJNA45811, CP002095	[44]	2011
BCG Korea 1168P	Roche 454 (GS-FLX) and Illumina (HiSeq) sequencing, **gap closure via PCR and primer walking**	IV	yes (2x)	PRJNA170028, CP003900	[45]	2013
BCG Russia 368	Roche 454 (GS Junior) sequencing on **a shotgun and a 3 kb paired-end library, gap closure via PCR and Sanger sequencing**	I	yes (2x)	PRJNA256163, CP009243	[46, 47]	2014
BCG 3281 (clinical isolate)	Roche 454 (GS-FLX) and Illumina (Hiseq2500) sequencing, **gap closure via PCR**	III	yes	PRJNA251957, CP008744	[27]	2015
BCG Russia BCG-1	Roche 454 (GS-FLX) sequencing of **a** **shotgun library**, Ion Torrent PGM sequencing of **a mate-pair library**	I	yes (2x)	PRJNA306822, CP013741	[48]	2016
BCG Danish 1331 (07/270)	PacBio (RSII) (long read) sequencing (235x coverage) and Illumina (MiSeq) (short read) sequencing	III	yes	PRJNA494982, CP039850	this study	2019

For each strain, we indicated the used method to create the assembled genome, the type of DU2 present in the strain, whether the DU2 was resolved in the genome assembly, the BioProject and genome assembly accession number, the reference to the study in which genome assembly method was published and the year of publication. In the ‘method’ description, we have put labor/capital-intensive aspects in bold, illustrating that our approach using solely massive parallel sequencing, is the only one that provides both high per-bp accuracy (allowing for SNP calling) and complete resolution of the assembly across large repeat regions

## Conclusions

Our data highlight the importance of characterizing our BCG vaccine strains, as more variability exists among these strains than was thought. The availability of the complete reference genome for BCG Danish 1331 as well as the associated genome analysis workflow, now permits full genomic characterization of (engineered) TB vaccine strains, which should contribute to more consistent manufacturing of this highly cost-effective vaccine that protects the world’s newborns from disseminated TB, and that is used as a basic chassis for improved TB vaccine design.

## Methods

### Mycobacterial strains, gDNA and reference genomes

The strains used include the *M. bovis* BCG Danish 1331 sub-strain (1^st^ WHO Reference Reagent, 07/270, National Institute for Biological Standards and Control (NIBSC), Hertfordshire), the BCG Pasteur 1173 strain (ATCC®35734™, ATCC, Manassas), the streptomycin-resistant BCG Pasteur 1721 strain [49] (*RpsL*: K43R; a gift of Dr. P. Sander, Institute for Medical Microbiology, Zürich). From the Danish 1331 strain, a *sapM* knockout (KO) strain was constructed (detailed procedure of the strain construction can be found in Additional file 1: Methods). Strains were grown in Middlebrook 7H9 broth (Difco) supplemented with 0.05% Tween-80 and Middlebrook OADC (Becton Dickinson). Preparation of genomic DNA (gDNA) from mycobacterial strains was performed as previously described [50]. As reference genomes, *M. tb* H37Rv (NC_000962.3 [51]), *M. bovis* AF2122_97 (NC_002945.4 [52]) and BCG Pasteur 1173P2 (NC_008769.1 [53]) were used.

### Whole genome sequencing of BCG Danish 1331 WT and *sapM* KO strain

For PacBio SMRT sequencing, the gDNA was sheared using a Megaruptor device (large hydropore, Megaruptor, Diagenode, shearing size 35 kb), used for PacBio SMRT library preparation (SMRTbell Temp Prep Kit 1.0, Pacific Biosciences). Size selection was done on a BluePippin device (0.75% DF marker S1 high-pass 15-20 kb, Sage Science). The prepared samples were sequenced on a PacBio RSII instrument (DNA/Polymerase Binding Kit P6 v2, DNA Sequencing Kit 4.0 v2, Pacific Biosciences), the mean read length was 13.7 kb. One SMRT-cell was used for the KO sample (229x coverage) and 2 SMRT-cells were run for the WT sample (140x and 95x coverage). For Illumina sequencing, libraries were prepared with the Nextera DNA Library Preparation kit and sequenced on an Illumina MiSeq instrument (MiSeq Reagent Kit v2 Nano, PE250 (paired end 250 bp), 500 Mb), with an average of 55-56x coverage per genome.

### Genome assembly and analysis

Illumina reads were quality-filtered and adapter sequences were trimmed (Trimmomatic v0.36 [54]), after which overlapping paired-end reads were merged into single reads (BBMerge v36.69 [55]). PacBio read sequences were corrected using the high quality Illumina reads (Lordec v0.6 [56]). The unmerged and merged Illumina reads were assembled into a draft assembly (SPAdes v3.9.0 [57]). The draft assembly was scaffolded using the corrected PacBio reads (SSPACE-LongRead v3.0 [58]). Finally, gaps in the scaffold were closed (GapFiller v1.10 [59]) and the assembly was improved (Pilon v1.20 [60]), both using the trimmed Illumina reads.

The exact sequence of the DU1 region was based on a second round of local de novo assembly (SPAdes v3.9.0 [57]) using soft-clipped Illumina reads surrounding the draft DU1 region where the Illumina read coverage is more than two times higher than the background coverage. The DU2 repeat was resolved by comparing the SPAdes assembly with the assembly from HINGE (v201705) [61], where the R1 and R2 regions have been separated. The junction sequences of DU1 and DU2 were further confirmed by aligning uniquely mapped PacBio reads and the results were always consistent with PCR and Sanger sequencing.

Annotation was done by combining an automatic gene prediction program with heuristic models (GeneMark.hmm [62]) and the existing *M. bovis* BCG Pasteur and *M. tb* reference [51] gene models (GMAP [63] and TBLASTN [64]) along with UniProt database [65] (BLASTP [64]). Non-coding RNA were predicted (tRNAScan-SE [66] and Infernal [67]). The assigned annotations were manually checked (Artemis [68] and CLC Main Workbench 8 [69], e.g. correct start codon), by comparative analysis with the 3 reference genomes for *M. tb* [51], *M. bovis* [52] and *M. bovis* BCG Pasteur [53], as listed above. Inconsistencies in the annotation and/or assembly were analyzed in detail and/or verified by PCR and Sanger Sequencing.

A probabilistic variant analysis was performed by mapping the BBmerged Illumina reads to the BCG Pasteur reference genome (BWA-MEM [70]) and calling variants by GATK UnifiedGenotyper [71] (Count ≥10 & Variant Probability > 0.9), whereafter variant annotations and functional effect prediction were carried out with SnpEff and SnpSift [72]. The orthologous relationships between *M. tb*, *M. bovis* BCG Pasteur and BCG Danish WT and *sapM* KO were investigated, the proteins of strains (*M. tb* H37Rv [51], BCG Pasteur 1173P2 [53], BCG Danish WT and *sapM* KO (this study)) were searched using all-against-all with BLASTP [64], after which the result was analyzed by TribeMCL [73] and i-ADHoRe 3.0 [74] based on the genome synteny information (Additional file 3: Table S9).

To validate the detection of the DU1, the DU1 duplication region was reanalyzed in published genome data [12, 15–20]. Probes on tiling array or Illumina short sequencing reads were mapped to the *M. tb* reference strain [48] (BWA-MEM [70]). The tilling array data were directly compared by the intensity ratio between H37Rv and the sampled strains (ratio = strain / H37Rv). A ratio larger than one was considered as a duplication in the sampled strain. The DU1 duplications in the Illumina data were detected by cn.mops [75]. In brief, cn.mops first took all aligned BAM files (BWA-MEM) and normalized the mappable read counts to make it compatible across all samples in the comparison. A mixture of Poisson model was then used to compare read counts for each genomic position (bin size 500 bp) across all samples. A mixture of Poisson model will not be affected by read count variations along the chromosomes caused by technical or biological noise, since a separate model is constructed at each position. Using a Bayesian approach, read counts and the noise across samples were decomposed by an expectation maximization algorithm into integer copy numbers (with confidence intervals).

In Fig. 1 a graphical overview of the performed genome analysis pipeline is given. All presented next-generation sequencing data were integrated in an online genome browser (JBrowse) [76].

### PCR analysis, gel electrophoresis and sanger sequencing

PCR (GoTaq®Green, Promega) was performed on gDNA using primers listed in Additional file 1: Table S1 and S4. PCR products were run on a 1.2% agarose gel, stained with Midori Green and visualized under ultraviolet light. To confirm the single nucleotide polymorphisms (SNPs), regions of interest were amplified (Phusion High-Fidelity DNA Polymerase, NEB) from gDNA with primers listed in Additional file 1: Table S5. The resulting PCR products were purified (AMPure XP beads) and Sanger sequenced with (a) nested primer(s) (Additional file 1: Table S1 and S5).

### Copy number profiling via qPCR

Real-time quantitative PCR was done on a LightCycler 480 (Roche Diagnostics) using the SensiFast SYBR-NoRox kit (Bioline) in quadruplicate for each gDNA sample using primers listed in Additional file 1: Table S6. Determination of the average relative quantities was performed using the qbasePLUS software (Biogazelle). All results were normalized using the reference genes 16S rRNA, *nuoG* and *mptpB*.

## Additional files

Additional file 1: Supplementary Methods, **Figures S1-S3** and **Tables S1-S7**. (DOCX 513 kb)

Additional file 2: **Table S8.** Distribution of regions of difference, deletions and tandem duplications (DU1 and DU2) in the different BCG strains compared to *M. bovis* AF2122_97 (NC_002945.4). (XLSX 14 kb)

Additional file 3: **Table S9.** Ortholog of mycobacterial genes between *M. tb* H37Rv, *M. bovis* BCG Pasteur, *M. bovis* BCG Danish WT and *sapM* KO. (XLSX 880 kb)

## Data Availability

The raw sequencing data (raw Illumina and PacBio reads, and PacBio base modification files) generated by this study for the BCG Danish 1331 WT and *sapM* KO strain, the complete genome assemblies have been submitted to NCBI under BioProject PRJNA494982 [21]. The genome annotations were deposited on the Figshare data repository with DOI 10.6084/m9.figshare.c.4489496 [77]. The publicly available datasets we analyzed during the study are available in the CIBEX database with identifier CBX70 [15] or in the NCBI repository with identifiers PRJNA63833 [16], PRJNA63835 [17], PRJNA63837 [18], PRJNA63839 [19], PRJEB8560 [12], and PRJNA506333 [20]. To maximize the community accessibility of these resources, we have integrated all of the presented next-generation sequencing data in an online genome browser (JBrowse) available from the website of YCL [76]. The previously published mycobacterial reference genomes that we consulted during the study are available from NCBI [51–53]. The data (other than the next-generation sequencing data) that support the findings of this study are available on request from the corresponding author NC.

## Abbreviations

BCG: Bacillus Calmette-Guérin
CDS: Coding sequence
gDNA: Genomic DNA
KO: Knockout
*M. bovis*: *Mycobacterium bovis*
*M. tb*: *Mycobacterium tuberculosis*
NIBSC: National Institute for Biological Standards and Control
RD: Region of difference
SNP: Single nucleotide polymorphism
TB: Tuberculosis
WHO: World Health Organization
WT: Wild type

## References

[1] Lugosi L (1992). Theoretical and methodological aspects of BCG vaccine from the discovery of Calmette and Guérin to molecular biology. A review. Tuber Lung Dis.

[2] Ritz N, Hanekom WA, Robins-Browne R, Britton WJ, Curtis N (2008). Influence of BCG vaccine strain on the immune response and protection against tuberculosis. FEMS Microbiol Rev.

[3] Zhang L, Ru H, Chen F, Jin C, Sun R, Fan X (2016). Variable virulence and efficacy of BCG vaccine strains in mice and correlation with genome polymorphisms. Mol Ther.

[4] Ho MM, Markey K, Rigsby P, Hockley J, Corbel MJ (2011). Report of an international collaborative study to establish the first WHO reference reagents for BCG vaccines of three different sub-strains. Vaccine..

[5] Zheng Y, Naguib YW, Dong Y, Shi Y, Bou S, Cui Z (2015). Applications of bacillus Calmette–Guerin and recombinant bacillus Calmette–Guerin in vaccine development and tumor immunotherapy. Expert Rev Vaccines.

[6] Nieuwenhuizen NE, Kaufmann SHE. Next-generation vaccines based on Bacille Calmette–Guérin. Front Immunol. 2018. 10.3389/fimmu.2018.00121. 10.3389/fimmu.2018.00121 PMC5807593 29459859

[7] Pan Y., Yang X., Duan J., Lu N., Leung A. S., Tran V., Hu Y., Wu N., Liu D., Wang Z., Yu X., Chen C., Zhang Y., Wan K., Liu J., Zhu B. (2011). Whole-Genome Sequences of Four Mycobacterium bovis BCG Vaccine Strains. Journal of Bacteriology.

[8] Abdallah AM, Hill-Cawthorne GA, Otto TD, Coll F, Guerra-Assunção JA, Gao G (2015). Genomic expression catalogue of a global collection of BCG vaccine strains show evidence for highly diverged metabolic and cell-wall adaptations. Sci Rep.

[9] Seki Masaaki, Honda Ikuro, Fujita Isao, Yano Ikuya, Yamamoto Saburo, Koyama Akira (2009). Whole genome sequence analysis of Mycobacterium bovis bacillus Calmette–Guérin (BCG) Tokyo 172: A comparative study of BCG vaccine substrains. Vaccine.

[10] Bedwell J, Kairo SK, Behr MA, Bygraves JA (2001). Identification of substrains of BCG vaccine using multiplex PCR. Vaccine..

[11] Brosch R, Gordon SV, Garnier T, Eiglmeier K, Frigui W, Valenti P (2007). Genome plasticity of BCG and impact on vaccine efficacy. Proc Natl Acad Sci.

[12] Abdallah AM, Hill-Cawthorne GA, Otto TD, Coll F, Guerra-Assunção JA, Gao G (2015). A genomic sequence and expression diversity catalogue of BCG (accession: PRJEB8560, ID: 294541). BioProject database.

[13] Brosch Roland, Gordon Stephen V., Buchrieser Carmen, Pym Alexander S., Garnier Thierry, Cole Stewart T. (2000). Comparative Genomics Uncovers Large Tandem Chromosomal Duplications inMycobacterium bovisBCG Pasteur. Yeast.

[14] Leung AS, Tran V, Wu Z, Yu X, Alexander DC, Gao GF (2008). Novel genome polymorphisms in BCG vaccine strains and impact on efficacy. BMC Genomics.

[15] Leung AS, Tran V, Wu Z, Yu X, Alexander DC, Gao GF (2008). Microarray data 13 BCG strains. CIBEX database.

[16] Pan Y, Yang X, Duan J, Lu N, Leung AS, Tran V. *Mycobacterium tuberculosis* variant bovis BCG str. China, whole genome shotgun sequencing project. BioProject database. 2011. https://www.ncbi.nlm.nih.gov/bioproject/PRJNA63833.

[17] Pan Y, Yang X, Duan J, Lu N, Leung AS, Tran V. *Mycobacterium tuberculosis* variant bovis BCG str. ATCC 35733 (Denmark 1331), whole genome shotgun sequencing project. BioProject database. 2011. https://www.ncbi.nlm.nih.gov/bioproject/PRJNA63835.

[18] Pan Y, Yang X, Duan J, Lu N, Leung AS, Tran V. *Mycobacterium tuberculosis* variant bovis BCG str. ATCC 35740 (Russia), whole genome shotgun sequencing project. BioProject database. 2011. https://www.ncbi.nlm.nih.gov/bioproject/PRJNA63837.

[19] Pan Y, Yang X, Duan J, Lu N, Leung AS, Tran V. *Mycobacterium tuberculosis* variant bovis BCG str. ATCC 35743 (Tice), whole genome shotgun sequencing project. BioProject database. 2011. https://www.ncbi.nlm.nih.gov/bioproject/PRJNA63839.

[20] Festjens N, Vandewalle K, Houthuys E, Plets E, Vanderschaeghe D, Borgers K. *Mycobacterium tuberculosis* variant bovis BCG, Pasteur 1721, raw sequence reads (ID: PRJNA506333). BioProject database. 2019. http://www.ncbi.nlm.nih.gov/bioproject/506330.

[21] Borgers K, Ou JY, Zheng PX, Tiels P, Van Hecke A, Plets E, et al. *Mycobacterium tuberculosis *variant bovis, genome sequencing and assembly (ID: PRJNA494982). BioProject database. 2019. http://www.ncbi.nlm.nih.gov/bioproject/494982

[22] Sadagopal S, Braunstein M, Hager CC, Wei J, Daniel AK, Bochan MR (2009). Reducing the activity and secretion of microbial antioxidants enhances the immunogenicity of BCG. PLoS One.

[23] Brosch R., Gordon S. V., Marmiesse M., Brodin P., Buchrieser C., Eiglmeier K., Garnier T., Gutierrez C., Hewinson G., Kremer K., Parsons L. M., Pym A. S., Samper S., van Soolingen D., Cole S. T. (2002). A new evolutionary scenario for theMycobacterium tuberculosiscomplex. Proceedings of the National Academy of Sciences.

[24] Mahairas G G, Sabo P J, Hickey M J, Singh D C, Stover C K (1996). Molecular analysis of genetic differences between Mycobacterium bovis BCG and virulent M. bovis. Journal of Bacteriology.

[25] Zhang W, Zhang Y, Zheng H, Pan Y, Liu H, Du P (2013). Genome sequencing and analysis of BCG vaccine strains. PLoS One.

[26] Magdalena J, Supply P, Locht C. Specific differentiation between *Mycobacterium bovis *BCG and virulent strains of the *Mycobacterium tuberculosis* complex. J Clin Microbiol. 1998;36:2471–6. 10.1128/jcm.36.9.2471-2476.1998 PMC105146 9705376

[27] Li Xuming, Chen Liping, Zhu Yongqiang, Yu Xia, Cao Jun, Wang Rui, Lv Xinyan, He Jin, Guo Aizhen, Huang Hairong, Zheng Huajun, Liu Siguo (2015). Genomic Analysis of a Mycobacterium Bovis Bacillus Calmette-Guérin Strain Isolated from an Adult Patient with Pulmonary Tuberculosis. PLOS ONE.

[28] Bottai D, Brosch R (2016). The BCG strain Pool: diversity matters. Mol Ther.

[29] Abdallah AM, Behr MA. Evolution and strain variation in BCG. In: Strain variation in the *Mycobacterium tuberculosis* complex: its role in biology, epidemiology and control: Springer; 2017. p. 155–69. 10.1007/978-3-319-64371-7_8 29116634

[30] Leonard AC, Grimwade JE (2011). Regulation of DnaA assembly and activity: taking directions from the genome. Annu Rev Microbiol.

[31] Salazar Leirla, Fsihi Hafida, Rossi Edda, Riccardi Giovanna, Rios Carmen, Cole Stewart T., Takiff Howard E. (1996). Organization of the origins of replication of the chromosomes of Mycobacterium smegmatis, Mycobacterium leprae and Mycobacterium tuberculosis and isolation of a functional origin from M. smegmatis. Molecular Microbiology.

[32] Ogasawara N, Moriya S, Yoshikawa H (1991). Initiation of chromosome replication: structure and function of oriC and DnaA protein in eubacteria. Res Microbiol.

[33] Zakrzewska-Czerwińska Jolanta, Nardmann Judith, Schrempf Hildgund (1994). Inducible transcription of the dnaA gene from Streptomyces lividans 66. MGG Molecular & General Genetics.

[34] Rajagopalan M, Qin M H, Nash D R, Madiraju M V (1995). Mycobacterium smegmatis dnaA region and autonomous replication activity. Journal of Bacteriology.

[35] Rajagopalan Malini, Dziedzic Renata, Al Zayer Maha, Stankowska Dorota, Ouimet Marie-Claude, Bastedo D. Patrick, Marczynski Gregory T., Madiraju Murty V. (2010). Mycobacterium tuberculosisOrigin of Replication and the Promoter for Immunodominant Secreted Antigen 85B Are the Targets of MtrA, the Essential Response Regulator. Journal of Biological Chemistry.

[36] Mycobrowser. https://mycobrowser.epfl.ch/. Accessed 21 Jan 2019.

[37] Lugosi L (1992). Theoretical and methodological aspects of BCG vaccine from the discovery of Calmette and Guerin to molecular biology. A review. Tuber Lung Dis.

[38] Song Houhui, Sandie Reatha, Wang Ying, Andrade-Navarro Miguel A., Niederweis Michael (2008). Identification of outer membrane proteins of Mycobacterium tuberculosis. Tuberculosis.

[39] Griffin Jennifer E., Gawronski Jeffrey D., DeJesus Michael A., Ioerger Thomas R., Akerley Brian J., Sassetti Christopher M. (2011). High-Resolution Phenotypic Profiling Defines Genes Essential for Mycobacterial Growth and Cholesterol Catabolism. PLoS Pathogens.

[40] DeJesus MA, Gerrick ER, Xu W, Park SW, Long JE, Boutte CC, et al. Comprehensive essentiality analysis of the *Mycobacterium tuberculosis* genome via saturating transposon mutagenesis. mBio. 2017;8:e02133–16. 10.1128/mBio.02133-16 PMC5241402 28096490

[41] Glenn TC (2011). Field guide to next-generation DNA sequencers. Mol Ecol Resour.

[42] Vollger MR, Dishuck PC, Sorensen M, Welch AE, Dang V, Dougherty ML (2019). Long-read sequence and assembly of segmental duplications. Nat Methods.

[43] Gomes L. H. F., Otto T. D., Vasconcellos E. A., Ferrao P. M., Maia R. M., Moreira A. S., Ferreira M. A., Castello-Branco L. R. R., Degrave W. M., Mendonca-Lima L. (2011). Genome Sequence of Mycobacterium bovis BCG Moreau, the Brazilian Vaccine Strain against Tuberculosis. Journal of Bacteriology.

[44] Orduña P, Cevallos MA, de León SP, Arvizu A, Hernández-González IL, Mendoza-Hernández G, et al. Genomic and proteomic analyses of *Mycobacterium bovis* BCG Mexico 1931 reveal a diverse immunogenic repertoire against tuberculosis infection. BMC Genomics. 2011;12:493. 10.1186/1471-2164-12-493 PMC3199284 21981907

[45] Joung SM, Jeon SJ, Lim YJ, Lim J-S, Choi B-S, Choi I-Y, et al. Complete genome sequence of *Mycobacterium bovis *BCG Korea, the Korean vaccine strain for substantial production. Genome Announc. 2013;1:e0006913. 10.1128/genomeA.00069-13 PMC3622960 23516200

[46] Kunda MS, Voronina OL, Aksenova EI, Semenov AN, Ruzhova N, Lunin VG, Troitsky A, Rusin L, Petrov N (2014). Analyzing of the BCG substrains diversity formed by the human influence. Molecular Phylogenetics: contributions to the 4th Moscow international conference “molecular Phylogenetics” (MolPy-4).

[47] Voronina OL, Kunda MS, Aksenova EI, Semenov AN, Ryzhova NN, Lunin VG, et al. Mosaic structure of *Mycobacterium bovis* BCG genomes as a representation of phage sequences’ mobility. BMC Genomics. 2016;17 Suppl 14. 10.1186/s12864-016-3355-1. 10.1186/s12864-016-3355-1 PMC5249017 28105923

[48] Sotnikova EA, Shitikov EA, Malakhova MV, Kostryukova ES, Ilina EN, Atrasheuskaya AV, et al. Complete genome sequence of *Mycobacterium bovis* strain BCG-1 (Russia). Genome Announc. 2016;4. 10.1128/genomeA.00182-16. 10.1128/genomeA.00182-16 PMC4816620 27034492

[49] Master Sharon S., Rampini Silvana K., Davis Alexander S., Keller Christine, Ehlers Stefan, Springer Burkhard, Timmins Graham S., Sander Peter, Deretic Vojo (2008). Mycobacterium tuberculosis Prevents Inflammasome Activation. Cell Host & Microbe.

[50] Vandewalle K, Festjens N, Plets E, Vuylsteke M, Saeys Y, Callewaert N. Characterization of genome-wide ordered sequence-tagged *Mycobacterium* mutant libraries by Cartesian pooling-coordinate sequencing. Nat Commun. 2015;6:7106. 10.1038/ncomms8106 PMC4432585 25960123

[51] Cole ST, Brosch R, Parkhill J, Garnier T, Churcher C, Harris D, et al. *Mycobacterium tuberculosis* H37Rv, complete genome. NCBI Nucleotide Database. 2017. NC_000962.3.

[52] Malone MK. *Mycobacterium bovis *AF2122/97 genome assembly. NCBI Nucleotide Database. 2017. NC_002945.4.

[53] Brosch R, Gordon SV, Garnier T, Eiglmeier K, Frigui W, Valenti P, et al. *Mycobacterium bovis* BCG Pasteur 1173P2, complete genome. NCBI Nucleotide Database. 2017. NC_008769.1.

[54] Bolger AM, Lohse M, Usadel B (2014). Trimmomatic: a flexible trimmer for Illumina sequence data. Bioinformatics..

[55] Bushnell B, Rood J, Singer E (2017). BBMerge - accurate paired shotgun read merging via overlap. PLoS One.

[56] Salmela L, Rivals E (2014). LoRDEC: accurate and efficient long read error correction. Bioinformatics..

[57] Bankevich A, Nurk S, Antipov D, Gurevich AA, Dvorkin M, Kulikov AS (2012). SPAdes: a new genome assembly algorithm and its applications to single-cell sequencing. J Comput Biol.

[58] Boetzer M, Pirovano W (2014). SSPACE-LongRead: scaffolding bacterial draft genomes using long read sequence information. BMC Bioinformatics..

[59] Nadalin F, Vezzi F, Policriti A (2012). GapFiller: a de novo assembly approach to fill the gap within paired reads. BMC Bioinformatics.

[60] Walker BJ, Abeel T, Shea T, Priest M, Abouelliel A, Sakthikumar S (2014). Pilon: an integrated tool for comprehensive microbial variant detection and genome assembly improvement. PLoS One.

[61] Kamath GM, Shomorony I, Xia F, Courtade TA, Tse DN (2017). HINGE: long-read assembly achieves optimal repeat resolution. Genome Res.

[62] Besemer J (2001). GeneMarkS: a self-training method for prediction of gene starts in microbial genomes. Implications for finding sequence motifs in regulatory regions. Nucleic Acids Res.

[63] Wu TD, Nacu S (2010). Fast and SNP-tolerant detection of complex variants and splicing in short reads. Bioinformatics..

[64] Altschul SF, Gish W, Miller W, Myers EW, Lipman DJ (1990). Basic local alignment search tool. J Mol Biol.

[65] The UniProt Consortium (2017). UniProt: the universal protein knowledgebase. Nucleic Acids Res.

[66] Lowe TM, Eddy SR (1997). tRNAscan-SE: a program for improved detection of transfer RNA genes in genomic sequence. Nucleic Acids Res.

[67] Nawrocki EP, Eddy SR (2013). Infernal 1.1: 100-fold faster RNA homology searches. Bioinformatics..

[68] Carver T, Harris SR, Berriman M, Parkhill J, McQuillan JA (2012). Artemis: an integrated platform for visualization and analysis of high-throughput sequence-based experimental data. Bioinformatics..

[69] Qiagen. CLC Main Workbench version 8. https://www.qiagenbioinformatics.com/. Accessed 3 Oct 2018.

[70] Li H. Aligning sequence reads, clone sequences and assembly contigs with BWA-MEM. arXiv. 2013; arXiv:1303.3997.

[71] McKenna A, Hanna M, Banks E, Sivachenko A, Cibulskis K, Kernytsky A (2010). The genome analysis toolkit: a MapReduce framework for analyzing next-generation DNA sequencing data. Genome Res.

[72] Cingolani P, Platts A, Wang LL, Coon M, Nguyen T, Wang L (2012). A program for annotating and predicting the effects of single nucleotide polymorphisms, SnpEff. Fly (Austin).

[73] van Dongen S, Abreu-Goodger C (2012). Using MCL to extract clusters from networks. Methods Mol Biol.

[74] Proost S, Fostier J, De Witte D, Dhoedt B, Demeester P, Van de Peer Y (2012). I-ADHoRe 3.0--fast and sensitive detection of genomic homology in extremely large data sets. Nucleic Acids Res.

[75] Klambauer G, Schwarzbauer K, Mayr A, Clevert D-A, Mitterecker A, Bodenhofer U (2012). Cn. MOPS: mixture of Poissons for discovering copy number variations in next-generation sequencing data with a low false discovery rate. Nucleic Acids Res.

[76] Bioinformatics & Computational Genomics lab. *M. bovis* BCG Danish genome resources. http://bcg.abrc.sinica.edu.tw/resources/mbovis-bcg-danish/. Accessed 20 May 2019.

[77] Borgers K, Ou JY, Zheng PX, Tiels P, Van Hecke A, Plets E, et al. Reference genome and comparative genome analysis for the WHO reference strain for *Mycobacterium bovis *BCG Danish, the present tuberculosis vaccine. Genome assemblies and annotations. Figshare. 2019. 10.6084/m9.figshare.c.4489496. Accessed 16 May 2019. 10.1186/s12864-019-5909-5 PMC6615170 31286858

