# Supplementary material for: Reference genome and comparative genome analysis for the WHO reference strain for Mycobacterium bovis BCG Danish, the present tuberculosis vaccine
Source: BMC Genomics. 2019 Jul 8;20:561. doi: 10.1186/s12864-019-5909-5 (PMC6615170; doi:10.1186/s12864-019-5909-5)
Supplement: Supplementary file 1 — Supplementary Methods, Figures S1-S3 and Tables S1-S7. (DOCX 513 kb) [file 12864_2019_5909_MOESM1_ESM.docx]

**Additional file 1: Supplementary Methods, Figures S1-S2 and Tables S1-S7**

**SUPPLEMENTARY METHODS**

**Generation of *M. bovis* BCG Danish 1331 *sapM* KO**

A *sapM* KO construct was made with the p2NIL and pGOAL17 vectors[1]. Hereto, a 5’ and 3’ sequence part of *sapM* of 700 bp was amplified by PCR from gDNA of *M. bovis* BCG (5’ Fw primer: ctgcagggctggtgggtttgctcgtcg, 5’ Rv primer: attaccctgttatccctacggcgaacgcctgggccatc, 3’ Fw primer: tagggataacagggtaattagccgccgtcgctattctgtg, 3’ Rv primer aagcttctcgtcgtcggactcggccg). In the 5’ Rv primer a stop codon was inserted to ensure that a truncated *sapM* is formed. The 5’ and 3’ part were fused by performing a PCR with the 5’ Fw primer and 3’ Rv primer. The resulting fragment was cut with PstI and HindIII and ligated in the p2NIL vector cut with the same restriction enzymes. This p2NILSapM700 vector was then cloned into the PacI site of the pGOAL17 vector to create the pGOAL17SapM700 vector (**Additional file 1: Figure S2**).

For the electroporation, *M. bovis* BCG Danish 1331 (containing two *sapM* loci) was grown to mid-log phase (OD_600_ 0.4-0.8) in 7H9-ADS-Tw medium (7H9 + 50 g/L bovine serum albumin fraction V, 20 g/L dextrose, 8.5 g/L NaCl, 0.05% Tween-80). 1.5% glycine was added into the culture the day before electroporation. On the day of electroporation, cells were harvested in 50 ml conical tubes at room temperature at 3700 rpm for 10 minutes. The cells were washed twice with 50 ml 0.05% Tween-80 (pre-warmed at 37°C), after which the cells were resuspended in 1 ml of 0.05% Tween-80. The UV-irradiated plasmid (100 mJ/cm^2^, to stimulate homologous recombination) was added to 200 µl of bacterial cells after which the electroporation was performed (GenePulser apparatus (Bio-Rad) set at 2500 mV, resistance 800 ohms, capacitance 25 µF). The cells were diluted with 1 ml of 7H9-ADS-Tw (pre-warmed at 37°C) after which 4 ml medium was added and the culture was placed overnight at 37°C. The culture was plated out on 7H10 with 50 µg/ml kanamycin and 50 µg/ml X-gal. The one blue colony (presence of *lacZ*) that was formed, was tested with colony PCR for the integration of the plasmid and grown in liquid 7H9 medium without kanamycin to stimulate a second homologous recombination, resulting in knocking out the *sapM* gene. To select for the clones that have lost the plasmid, the culture was plated on 7H10 + 2% sucrose + 50 µg/ml X-gal (presence of *sacB* which inhibits growth on sucrose medium). Several white colonies were tested for the absence of *sapM*, by means of PCR. One clone was selected for further work, which showed absence of SapM expression and thus lost both *sapM* loci. This was confirmed by PCR, Southern Blot, qPCR-RT analysis, SapM ELISA and phosphatase assay (**Additional file 1: Figure S3a-f**). The SapM ELISA and phosphatase assay were performed as described [2].

**Southern blot**

To verify if deletion of the *sapM* gene had occurred, genomic DNA of the strains was digested with PvuII. The digested samples were blotted to an Amersham Hybond-N+ membrane (GE Healthcare) by the neutral denaturing procedure (according to the manufacturer’s instructions). We hybridized the membranes with a DIG-labelled *sapM* probe, created by PCR amplifying a region overlapping with the 5’ end of *sapM* (primers GGCTGGTGGGTTTGCTCGTCG and TGCCAGACCCACTTGTGGGACA) using a DIG-labeled synthetic dNTP mix (Roche Life Sciences). The membrane was incubated with an anti-DIG-AP antibody (1:10,000) (Roche Life Sciences), washed twice with washing buffer (0.1 M of maleic acid, 0.15 M of NaCl and 0.3% Tween-20, pH 7.5) and developed with the Amersham CDP-Star substrate in detection buffer (1:100 dilution) (GE Healthcare). The luminescent signal was measured by exposure to an X-ray film. The expected Southern Blot band for the WT was 2206 bp and 1598 bp for the *sapM* KO (**Additional file 1: Figure S3a**).

RT-qPCR analysis

*M. bovis* BCG cultures (grown in standard 7H9 medium until an OD_600_ of 0.8 – 1.0) were centrifuged and the pellets were washed once with sterile water containing 0.5% Tween-80. The pellet was then resuspended in 500 µl of RLT buffer (RNeasy Mini Kit, Qiagen; supplemented with β-ME). The cells were disrupted with glass beads in a Retsch MM2000 bead beater at 4°C in screw-cap tubes (pre-baked at 150°C). After centrifugation (2 min, 13,000 rpm, 4°C), the supernatant was transferred to a fresh eppendorf tube. To recover the lysate trapped in between the beads, 800 µl of chloroform was added to the beads and centrifuged, after which the upper phase was transferred to the same eppendorf tube as before. Then, 1 volume of Acid Phenol/Chloroform (Ambion) was added, incubated for 2 minutes and centrifuged (5 min, 13,000 rpm, 4°C). The upper aqueous phase was transferred to a fresh eppendorf tube and this last step was repeated once. An equal volume of 70% ethanol was added and the sample was transferred to an RNeasy spin column (RNeasy Mini Kit, Qiagen). The kit manufacturer’s instructions were followed to purify the RNA. After elution in RNase-free water (30 µl), an extra DNase digestion was performed with DNaseI (10 U of enzyme, 50 µl reaction volume). Then an extra clean-up step was performed with the RNeasy Mini Kit (Qiagen). Finally, the RNA concentration was determined on a Nanodrop instrument.

cDNA was prepared from 1 μg of DNase-treated RNA using the iScript Synthesis Kit (BioRad) and a control reaction lacking reverse transcriptase was included for each sample. The RT-PCR program was as follows: 10 min at 25°C, 30 min at 42°C, 5 min at 85°C.

Real time quantitative PCR was done on a LightCycler 480 (Roche Diagnostics) using the SensiFast SYBR-NoRox kit (BioLine), in triplicate for each cDNA sample. All gene expression values were normalized using the geometric mean of the *gap* and *pgk* rRNA. Determination of amplification efficiencies and conversion of raw Cq values to normalized relative quantities (NRQ) was performed using the qbasePLUS software. Statistical analysis of the NRQs was done with the Prism6.04 software package using an unpaired t-test, we corrected for multiple comparisons using the Holm-Sidak method (alpha = 0.05).

Primers used in the experiment: *gap* (TGGGAGTTAACGACGACAAG and ACTCATCGTCGAGCACTTTG), *pgk* (GAAACCAGCAAGAACGATGA and AACAGGGTTGCGATGTCATA), *upp* (CGGGTCGCGGCTAAC and GGGCAGCGAGTCCAGATA), *sapM* (TGCGGCCCGGAACTTACAACGAGA and CAAGCGGATGGGTACGAGGTCAGC) and BCG_3376 (AAGTTCTTCAACGGCAATCC and GTGCTGATGATCTCGTCGAT).

**SUPPLEMENTAL FIGURES**

**
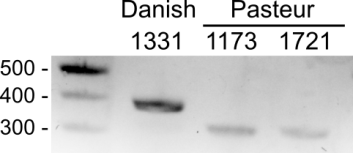
**

**Figure S1. Analysis of the SenX3-RegX3 region in BCG strains.** PCR was performed on gDNA of BCG Danish 1331, Pasteur 1173 and 1721 using primer pair 9. This primer set was originally designed by Bedwell *et al.* 2001 [3] to identify BCG substrains by multiplex-PCR. Dependent on the number of repeats of 77 bp in the SenX3-RegX3 region a different amplicon is formed; a 353 bp amplicon for 3 repeats and a 276 bp amplicon for 2 repeats.

**
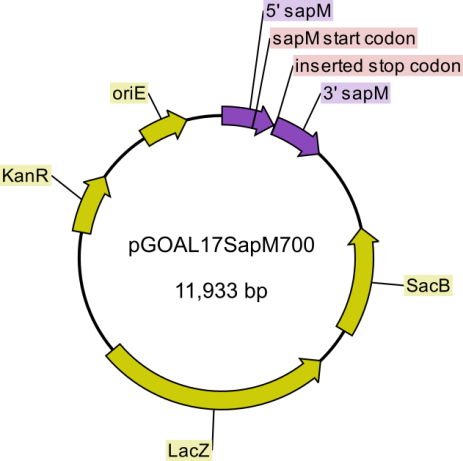
**

**Figure S2. Plasmid map of suicide vector pGOAL17SapM700.**

**
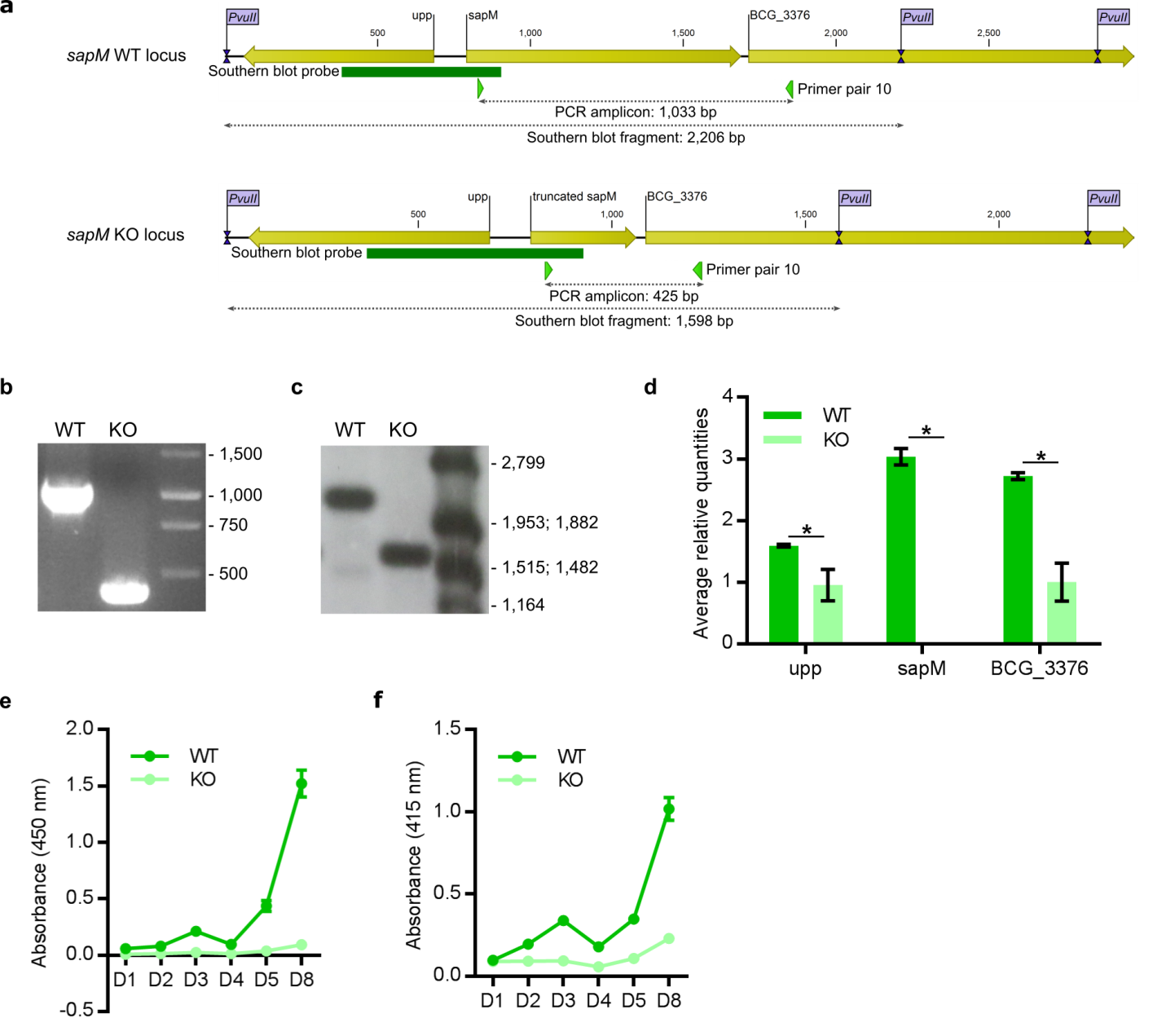
**

**Figure S3. Validation of the BCG Danish 1331 *sapM* KO on genome, mRNA and protein level**. **a)** Representation of the *sapM* WT and KO locus with indication of theoretical PCR amplicons and Southern blot fragments (PvuII digest and hybridization with Southern blot probe). **b)** PCR analysis of the *sapM* locus. WT and *sapM* KO gDNA were amplified with primer pair 10. **c)** Southern blot for the 5’ part of *sapM* of *M. bovis* BCG WT and *sapM* KO after *PvuII* digest. **d)** RT-PCR analysis of the *sapM* locus. RNA was prepared of cultures of biological triplicates of the *sapM* KO and parental strain. RT-PCR on the cDNA was performed using primer sets directed against *sapM* and the directly up- and downstream genes (*upp* and BCG_3376). The data presented here are averages (± SEM) of three biological replicates. (*: p < 0.05). **e-f)** Analysis of SapM protein quantity and enzymatic activity. Strains were subcultured from a mid-log culture to an OD_600_ of 0.1, and medium samples were collected for 8 days of subsequent growth of this culture. ELISA using an anti-SapM polyclonal antibody was performed to detect the SapM protein **(e)**. A phosphatase assay was performed using p-nitrophenyl phosphate as a substrate to detect secreted phosphatase activity, most of which is due to SapM in WT cultures **(f)**. The represented data are averages (± SD) of three technical replicates.

**SUPPLEMENTARY TABLES**

**Table S1 | Ambiguous regions that were locally reassembled and/or experimentally verified.**

| **Ambiguous regions** | **Local reassembly** | **Experimental verification** | **Result** |
| --- | --- | --- | --- |
| DU1 | yes, as described in M&M | PCR (see **Figure 1a**) and qPCR (see **Figure 1d**) | assembly was validated |
| DU2 | yes as described in M&M | PCR (see **Figure 1a**) | assembly was validated |
| high GC content region 1 "gttgccggccgccagaccattggcccccgcgcgagcaccggcgccgcccgacgcccccccccgcccccccccccccccccccccacccccgccagccccgccgtctccggcccggccgacataccccaatagtccagccgacccgccagcacccccggcgccgcccacgccgccgtttcc" | no | PCR and Sanger sequencing with primers KB291 and KB292 | sequence was changed to “gttgccggccgccagaccattggcccccgcgcgagcaccggcgccgcccgacgccccccccggctccgccagccccacccaggcccgggtcgccaccgtgaccgccggccccgccgtcaccggcggccagccaactgccaccgttgccgccggcaccgccgtcaccaggcgctccacccagccccccacccccgccagccccgccgtctccggcccggccgacataccccaatagtccagccgacccgccagcacccccggcgccgcccacgccgccgtttcc” |
| high GC content region 2 "gccggcgcgatccagcgctacaagctgaccgcggccacgcacgcgctgaagaagttgctgttcccttgggggggggggggggggggggggggggggggggggggggggggggggggggggggggcggatgagcgcctatcgccagccggtcgaaagatactggtgggcgaggcggcgttc" | no | PCR and Sanger sequencing with primers KB293 and KB294 | sequence was changed to “gccggcgcgatccagcgctacaagctgaccgcggccacgcacgcgctgaagaagttgctgttcccttgggggggggggggggggcggatgagcgcctatcgccagccggtcgaaagatactggtgggcgaggcggcgttc” |
| 120 bp deletion in *rpfA* only in Danish *sapM* KO | yes* | PCR with primer set KB295+KB296 and KB307+KB308 gave similar amplicons for all strains (Sanger sequencing was unsuccessful) | same situation in BCG Danish strains as in the Pasteur reference genome (no deletion in Danish *sapM* KO) |
| different assembly for PPE34 in Danish WT versus *sapM* KO | yes* | PCR and Sanger sequencing with primer sets KB297+KB298, KB299+KB300, KB309+KB310 and KB311+KB312 similar amplicons for all strains, Sanger sequencing of the first primer set was unsuccessful | same situation in BCG Danish strains as in the Pasteur reference genome |
| different assembly for PE_PGRS55 in Danish (WT and *sapM* KO) versus Pasteur | yes* | PCR with KB301+KB302, KB313+KB314 gave similar amplicons for all strains (Sanger sequencing was unsuccessful) | same situation in BCG Danish strains as in the Pasteur reference genome |
| different assembly for PE_PGRS54 in Danish WT versus *sapM* KO and versus Pasteur | yes* | PCR with primer sets KB303+KB304, KB305+KB306, KB315+KB316, KB317+KB318, KB305+KB319 and KB317+KB319 gave similar amplicons for the Danish strains (Sanger sequencing was unsuccessful) | both Danish strains have a 465 bp insertion compared to the Pasteur reference |
| different assembly for BCG_2100c in Danish WT versus *sapM* KO versus Pasteur | yes* | PCR and Sanger sequencing with primer set KB323+KB324 | sequences were changed to the sequences as determined by Sanger sequencing, however the exact length of the C/G-repeat could not be determined |
| different assembly for BCG_3898 in Danish (WT and *sapM* KO) (1 bp deletion) versus Pasteur | yes* | PCR and Sanger sequencing with primer sets KB325+KB326 | indeed 1 bp deletion in BCG Danish (WT and *sapM* KO) versus BCG Pasteur |
| different assembly for fadE19 in Danish *sapM* KO (1 bp deletion) versus Danish WT and Pasteur | yes* | no | same situation in Danish *sapM* KO as in Danish WT and Pasteur |
| different assembly for BCG_1373c and BCG_1374c in Danish WT (11 bp deletion) versus Danish *sapM* KO and Pasteur | yes* | no | same situation in Danish WT as in Danish *sapM* KO and Pasteur |
| different assembly for BCG_2527 in Danish WT (large deletion) versus Danish *sapM* KO and Pasteur | yes* | no | same situation in Danish WT as in Danish *sapM* KO and Pasteur |
| different assembly for aroE in Danish WT (deletion in C-repeat) versus Danish *sapM* KO and Pasteur | yes* | no | same situation in Danish WT as in Danish *sapM* KO and Pasteur |
| different assembly for PPE42 in Danish WT (deletion in C-repeat) versus Danish *sapM* KO and Pasteur | yes* | no | same situation in Danish WT as in Danish *sapM* KO and Pasteur |
| different assembly for PE_PGRS27-BCG_1514 in Danish WT (large deletion) versus Danish *sapM* KO and Pasteur | yes* | no | same situation in Danish WT as in Danish *sapM* KO and Pasteur |
| different assembly for BCG_2530c in Danish WT (1 bp substitution) versus Danish *sapM* KO and Pasteur | yes* | no | same situation in Danish WT as in Danish *sapM* KO and Pasteur |
| different assembly for PE_PGRS27-BCG_1514 in Danish WT versus Danish *sapM* KO and Pasteur | yes* | no | same situation in Danish WT as in Danish *sapM* KO and Pasteur |
| All ambiguous regions were located in GC-rich and/or highly repetitive regions, which are more difficult to sequence and assemble correctly.  Primer sequences are listed in **Table S7**. Both Danish strains (WT and *sapM* KO) and Pasteur strain were taken along in the experimental verification. M&M: Materials and Methods.  * local analysis of the PacBio and/or Illumina sequencing reads followed by reassembly of the region (for the last ambiguous regions we did not perform experimental verification if the PacBio and/or Illumina sequencing reads backed up the reassembly of the region) | | | |

**Table S2: Summary table of SNPs detected in *M. bovis* Danish 1331 WT and *sapM* KO compared to the Pasteur reference 1173P2 (NC_008679.1)** [4]**.**

| **Variants** | **Type** | **WT** | **KO** | **non-shared** |
| --- | --- | --- | --- | --- |
| SNPs | in intergenic region | 9 | 9 | 0 |
|  | synonymous | 9 | 9 | 0 |
|  | nonsynonymous, missense | 23 | 24 | 1 |
|  | nonsynonymous, nonsense | 1 | 1 | 0 |
|  | **total** | **42** | **43** | **1** |
| genes affected by SNPs | missense | 18 | 19 | 1 |
|  | nonsense | 1 | 1 | 0 |
|  | **total** | **19** | **20** | **1** |

**Table S3: SNPs detected in *M. bovis* Danish 1331 WT and *sapM* KO compared to the Pasteur reference 1173P2 (NC_008769.1)** [4]**.**

| **Nb** | **Position**  **in Pasteur** | **Pasteur** | **Danish** | | **Type** | **Variant** | **Affected gene** | **COG ID** | **COG category** | **SNP validation*** |
| --- | --- | --- | --- | --- | --- | --- | --- | --- | --- | --- |
|  |  |  | **WT** | **KO** |  |  |  |  |  |  |
| 1 | 68923 | A | G | G | non-synonymous, missense | p.Leu186Pro | BCG_0067c | NA | NA | successful |
| 2 | 118865 | T | C | C | non-synonymous, missense | p.Cys35Arg | BCG_0113 | NA | NA | successful |
| 3 | 190828 | C | T | T | synonymous | p.Ala21Ala | BCG_0167 | NA | NA | not performed |
| 4 | 370270 | C | T | T | in intergenic region | . | . | . | . | not performed |
| 5 | 623418 | C | T | T | non-synonymous, nonsense | p.Gln323* | galE2 | COG0451, COG1088 | [M] | successful |
| 6 | 705623 | G | A | A | synonymous | p.His392His | PE_PGRS7 | NA | NA | not performed |
| 7 | 705626 | G | C | C | synonymous | p.Ala391Ala | PE_PGRS7 | NA | NA | not performed |
| 8 | 759614 | T | C | C | non-synonymous, missense | p.Met184Val | echA3 | COG1024 | [I] | successful |
| 9 | 1324458 | T | C | C | non-synonymous, missense | p.Leu106Pro | narJ | COG2180 | [C],[P],[O] | successful |
| 10 | 1344671 | C | G | G | in intergenic region | . | . | . | . | not performed |
| 11 | 1344672 | G | C | C | in intergenic region | . | . | . | . | not performed |
| 12 | 1661300 | A | C | C | non-synonymous, missense | p.Val650Gly | PE_PGRS28 | NA | NA | no data (**) |
| 13 | 1661303 | A | T | T | non-synonymous, missense | p.Ile649Asn | PE_PGRS28 | NA | NA | no data (**) |
| 14 | 1661304 | T | C | C | non-synonymous, missense | p.Ile649Val | PE_PGRS28 | NA | NA | no data (**) |
| 15 | 1661306 | A | C | C | non-synonymous, missense | p.Ile648Ser | PE_PGRS28 | NA | NA | no data (**) |
| 16 | 1661307 | T | C | C | non-synonymous, missense | p.Ile648Val | PE_PGRS28 | NA | NA | no data (**) |
| 17 | 1661309 | T | C | C | non-synonymous, missense | p.Asp647Gly | PE_PGRS28 | NA | NA | no data (**) |
| 18 | 1661323 | G | C | C | synonymous | p.Gly642Gly | PE_PGRS28 | NA | NA | no data (**) |
| 19 | 1906675 | G | A | A | non-synonymous, missense | p.Asp119Asn | BCG_1714 | NA | NA | successful |
| 20 | 1937867 | G | T | T | non-synonymous, missense | p.Ala2Asp | PPE22 | COG5651 | [S] | successful |
| 21 | 2002385 | G | C | C | non-synonymous, missense | p.Arg129Gly | wag22b | NA | NA | no data (**) |
| 22 | 2143356 | T | G | G | in intergenic region | . | . | . | . | not performed |
| 23 | 2143357 | A | G | G | in intergenic region | . | . | . | . | not performed |
| 24 | 2442228 | G | A | A | synonymous | p.Gly23Gly | BCG_2215c | NA | NA | not performed |
| 25 | 2520484 | C | T | T | non-synonymous, missense | p.Arg260His | BCG_2284c | NA | NA | successful |
| 26 | 2619880 | T | C | C | non-synonymous, missense | p.Gln79Arg | hrcA | COG1420 | [K] | successful |
| 27 | 2734209 | G | A | A | non-synonymous, missense | p.Met18Ile | pepN | COG0308 | [E] | successful |
| 28 | 2764157 | T | A | A | synonymous | p.Thr177Thr | BCG_2507c | COG3903, COG2114 | [R], [T] | not performed |
| 29 | 2766103 | G | C | C | non-synonymous, missense | p.Ala804Gly | PE_PGRS43b | NA | NA | no data (**) |
| 30 | 2860207 | A | G | G | non-synonymous, missense | p.Cys68Arg | BCG_2594c | COG4129 | [S] | successful |
| 31 | 2976282 | T | C | C | non-synonymous, missense | p.Val46Ala | sigB | COG0568, COG1191 | [K] | successful |
| 32 | 3179819 | T | C | C | synonymous | p.Leu133Leu | ffh | COG0541, COG0552 | [U] | not performed |
| 33 | 3237056 | G | T | T | in intergenic region | . | . |  |  | not performed |
| 34 | 3315469 | A | G | G | non-synonymous, missense | p.Ile50Thr | ilvH | NA | NA | successful |
| 35 | 3443321 | G | A | A | non-synonymous, missense | p.Ser14Leu | PPE49a | COG5651 | [S] | successful |
| 36 | 3452980 | G | A | A | in intergenic region | . | . | . | . | not performed |
| 37 | 3606542 | C | T | T | synonymous | p.Asp208Asp | BCG_3301 | COG1804 | [I] | not performed |
| 38 | 3716536 | A | G | G | non-synonymous, missense | p.Val198Ala | icd1 | COG0538 | [C] | successful |
| 39 | 3907860 | A | G | G | non-synonymous, missense | p.Asn599Asp | PE_PGRS53 | NA | NA | successful |
| 40 | 3908180 | C | T | T | synonymous | p.Gly705Gly | PE_PGRS53 | NA | NA | no data (**) |
| 41 | 4061576 | C | G | G | in intergenic region | . | . | . | . | not performed |
| 42 | 4080517 | T | A | A | in intergenic region | . | . | . | . | not performed |
| 43 | 4359392 | A | A | C | non-synonymous, missense | p.Asn737Thr | BCG_3966 | NA | NA | successful |
| COG category: [C] Energy production and conversion, [E] Amino acid metabolism and transport, [I] Lipid metabolism, [K] Transcription, [M] Cell wall/membrane/envelop biogenesis, [O] Post-translational modification, protein turnover, chaperone functions, [P] Inorganic ion transport and metabolism, [R] General Functional Prediction only, [S] function Unknown, [T] Signal transduction mechanisms, [U] Intracellular trafficking, secretion, and vesicular transport.  NA: no corresponding COG category  *: SNP validation occurred via PCR and Sanger sequencing for all genes containing missense or nonsense SNPs.  **: PCR and/or Sanger sequencing was unsuccessful and therefore these SNPs could not be validated. All these regions are GC-rich or contain repeats.  Used primer sequences are indicated in **Table S4**. | | | | | | | | | | |

**Table S4. PCR primer pairs for confirmation of the genome assembly (pair 1-8), analysis of the SenX3-RegX3 region (pair 9) and validation of the KO engineering (pair 10).**

| **Primer set** | **Fw primer (5’ -> 3’)** | **Rv primer (5’ -> 3’)** | **Amplicon (bp)** |
| --- | --- | --- | --- |
| Pair 1 | GTTGTTTACCGTCCTCGG | GCCAGTCGAATCGTCATC | 804 |
| Pair 2 | GTCCAGCAGAAATGGCAG | GGTGACGCAGGTCTACAT | 502 |
| Pair 3 | CATCAAACTCGTCGCACA | GTTCGGGCGGTTCATATC | 675 |
| Pair 4 | CGAATCGCAGTTACCCTG | ACGGATGATCTCTGCCAT | 939 |
| Pair 5 | TTGCCAGGTGAAGGTAGT | CGTGGCTATCACTCCTCT | 928 |
| Pair 6 | TAACTCCAGAACGGGACC | ATCGACTATCCGCCACTT | 707 |
| Pair 7 | TAACAAGTCGGCTCCCTT | ATGGGATTGCCGTTGAAG | 1130 |
| Pair 8 | ACCGCACAAAGTTAAGAG | TCTCCATACCGATAGCTG | 987 |
| Pair 9 | GCGCGAGAGCCCGAACTGC | GCGCAGCAGAAACGTCAGC | 353 (3 repeats), 276 (2 repeats) |
| Pair 10 | AGAGACGCTCTCGAAGCCATACAGG | CCAGGGTATACCGTGCCTTGG | 1033 WT, 425 KO |

**Table S5. PCR primer pairs for confirmation of the SNP variants.**

| **SNP Nb**  (**Table S2**) | **Position** | **Fw PCR primer (5’ -> 3’)** | **Rv PCR primer (5’ -> 3’)** | **Nested Sanger sequencing primer(s) (5’ -> 3’)** |
| --- | --- | --- | --- | --- |
| 1 | 68923 | ACTGGGCTGCAGTTGGATGAAC | ACCTGCCGAACTGCTCACAGAC | CACTGATCTTCCACTGACGTCTCATC |
| 2 | 118865 | GCTATGGACGCCATGGACTACGAC | CGAGGACAGCAACTCACCGAC | GATTGTGAATTCGCGTCCGACAC |
| 5 | 623418 | AGGACAGCAGCAGTCGACGTC | CATTTGGATCGGACAGCAACGAC | ACTGCAGTTGCTGCACGAGC |
| 8 | 759614 | GATCAGGGACTGCGGATTGC | TATGGACGACGGCAAGGTCAAC | GACGCGAGATCACCGAACGAC |
| 9 | 1324458 | CTATGTCATTCCGACGTCGTACGC | CATGATCACCAGCAAGCTGCC | ATGTGGTCCAGCGATGAAGTTGC |
| 12 | 1661300 | CTATCTGGCGGTGGTGTTCTGC | AGTCGGTGGCAAAGGCAATAGC | GTAGTCAGATGGTTGGATCGCCAC |
| 13 | 1661303 | CTATCTGGCGGTGGTGTTCTGC | AGTCGGTGGCAAAGGCAATAGC | GTAGTCAGATGGTTGGATCGCCAC |
| 14 | 1661304 | CTATCTGGCGGTGGTGTTCTGC | AGTCGGTGGCAAAGGCAATAGC | GTAGTCAGATGGTTGGATCGCCAC |
| 15 | 1661306 | CTATCTGGCGGTGGTGTTCTGC | AGTCGGTGGCAAAGGCAATAGC | GTAGTCAGATGGTTGGATCGCCAC |
| 16 | 1661307 | CTATCTGGCGGTGGTGTTCTGC | AGTCGGTGGCAAAGGCAATAGC | GTAGTCAGATGGTTGGATCGCCAC |
| 17 | 1661309 | CTATCTGGCGGTGGTGTTCTGC | AGTCGGTGGCAAAGGCAATAGC | GTAGTCAGATGGTTGGATCGCCAC |
| 18 | 1661323 | CTATCTGGCGGTGGTGTTCTGC | AGTCGGTGGCAAAGGCAATAGC | GTAGTCAGATGGTTGGATCGCCAC |
| 19 | 1906675 | ATCCGTGTCGCACAATCCACAC | CTGCGAACCACATGCAGTGAC | ATCGTGCTGCCAATTGTCAGC |
| 20 | 1937867 | GATCAGCTCCGCTATTGCGCTC | GTTAACGCTGAATGCTCTGCAGTTC | GACCGAACACGTTATGCGTGACC |
| 21 | 2002385 | ATGTCCTGCTGCAGGTTGTGC | GCTGCTCAACTCGATCAACGC | CTTACCACCACCGAAGACAGTGC |
| 25 | 2520484 | AGACGCTGCCAGTTACGTGGTC | GAAGACGAGTTCGTGTGGTGTATGC | CAGTGCTCATCGACAATCGCAC |
| 26 | 2619880 | GCTCAGACGCCGTCTCATGAC | AGACGCCTGCTAGATGACGGTC | CTGAAAGCTTCTTGCCTTCCAGC |
| 27 | 2734209 | CCACACCATTGACATGGATCGTC | GATCAAGGCCACCAGATAGGTGC | CCATGCCTTCGCGGTATTGC |
| 29 | 2766103 | ACAGGAAGCGATCCGGATTCTC | CAACGGCGGCATTGGTATCAC | CAAGCCACCATTACCAGCGTC |
| 30 | 2860207 | GTTGGCAGCTTATCGCCACC | ATGGACTCGATCCGTGAGGTGATC | GTTCAGCTGCTGATGCAGTCGATC |
| 31 | 2976282 | CTCTCGAAGCCGGAGTGCAC | GAGCTCCTCATCGGTGGCTTC | GACTGATCTCGCTACTGATCGTGGC |
| 34 | 3315469 | CTGAATGATCGACAGGTCTGCGTC | AGATGACCAACCAGGAGCTGGC | GTGACACCGAGTGCTCGTCGTC |
| 35 | 3443321 | CTGAGCCTGCCCTAAGCCAGC | GACGGTATGGTCATCCGGTTTGATC | CTATCAACGACGCCAGCGACAC |
| 38 | 3716536 | GAAGTCGATGACTTCGGGAGTGC | GGACTCCCATGTCCAACGCAC | CAGCACCGACGTCATCAGGC |
| 39 | 3907860 | GATCGGTGGCCAGGGTACC | GTGATGGTGGTGCCCTGTGC | GTGATGGTGGTGCCCTGTGC, ACAACACCCTCAACCCCGACAC |
| 40 | 3908180 | GATCGGTGGCCAGGGTACC | GTGATGGTGGTGCCCTGTGC | GTGATGGTGGTGCCCTGTGC, ACAACACCCTCAACCCCGACAC |
| 43 | 4359392 | GTTGACCATCGATGACCGCAC | ATCAACCGCACAAGCAGTGGC | ATCAACCGCACAAGCAGTGGC, CTACACTCTGGCCACCGAGCAC |

**Table S6. qPCR primer pairs for copy number profiling.**

| **Genes** |  | **Fw primer (5’ -> 3’)** | **Rv primer (5’ -> 3’)** |
| --- | --- | --- | --- |
| ref genes | 16S rRNA | ATGACGGCCTTCGGGTTGTAA | CGGCTGCTGGCACGTAGTTG |
|  | *nuoG* | CTGCTGCTGATCAACCATCC | CCGTTAGACATTGCCTGGTT |
|  | *mptpB* | CCGCGTCATTTATCGACTGG | CGCATTCAAGAGGCTGCTAA |
| DU1 | *esxF* | CAGTTGAACCGCGAGATG | CTGGGCATTGTTAGGTTGC |
|  | BCG_3957c | GATCGTCACCGATATCTTCG | TCCAAACCAGGTGCAATG |
|  | *sigMa* | GCCAGGGTGAAGTGCTATG | CGGAACAACTGATCGAAGG |
|  | *trxB2* | TACCGACGCATTGATAGGAG | CAAAAGTCAACCAGCACAGG |
|  | *parB* | CCATCAGTGGTCATCAAACC | ATGTTGCTGAGCGTCTATCG |
|  | *rpmH* | GTTGCAATCAAGCACTGAGC | GTTCACGGTTTCCGCTTAC |
|  | *dnaN* | TGGCTGTTCGAGAACTGAAG | ACGACAAACGAACGTCAGAG |
|  | *recF* | CTTCGAGCTGTGTTGTTTGC | TCGCCAGATCATCCAGATAG |
|  | *gyrA* | GCTGATCCGCTATTACGTTG | ATCAGTGCAATGACCTCGTC |
|  | BCG_0012 | GTTGTGCATCAGCAAGACATC | CGACACAACGAGCTATCGAC |
|  | *pknA* | CATTGCGCTATCTCGTATCG | CGACTCAATTGGACGGAAC |
|  | BCG_0051 | ATTGGCGATGCTGTAGTCAC | CGAACTCGACATTCTCCATC |
| DU2 | *whiB7* | TCACACACAGTGTCTTGGCTAC | AGACCCCCAGACAAAGATTG |
|  | BCG_3268c | CTTTGAGAACGATGCTGTCC | TTGCTATCTCTTCCACGAAGG |
|  | BCG_3301 | AAACACTGGCAGAAGCTGTG | CCAGTGCCAATTCCAACTC |
|  | *atsB* | AACACCGGTTCGTTCAATG | CCGTATTGTTCGATCAGCTC |
|  | *upp* | CACGCTGCTGTTGATCTATG | GGGCAGCGAGTCCAGATA |
|  | *sapM* 5' | AGGGTATACCGTGCCTTGG | GTTCTCCTCCACCACGATG |
|  | *sapM* 3' | TGCGGCCCGGAACTTACAACGAGA | CAAGCGGATGGGTACGAGGTCAGC |
|  | BCG_3376 | AAGTTCTTCAACGGCAATCC | GTGCTGATGATCTCGTCGAT |
|  | *cdd* | GGAGCCTATGTGCCGTATTC | CGCACAAAGTCAAGCCATAC |

**Table S7. Primers for verification of the ambiguous regions.**

| **Primer** | **Primer sequence (5’ -> 3’)** |
| --- | --- |
| KB291 | CGTTACCTCCATCCTCACC |
| KB292 | GGCTGGACTATTGGGGTATGTC |
| KB293 | CAACTGCATGTTGTGCTACTC |
| KB294 | GACGACGTTCAGCACTACGAC |
| KB295 | TTCCAAATCGGTGGCAAGGC |
| KB296 | GTCAACGGCGAACCAGCAC |
| KB297 | TGATGTTGATCGGTCCCAAGC |
| KB298 | CATCGGTCCGATTGTGGTGC |
| KB299 | CGATTTCGATCGGGATGTTGATC |
| KB300 | TGGGCATTAACAGTTCCGGAGC |
| KB301 | CACCGGTACTGCAGGTGGC |
| KB302 | TGCTGCCTGCGAGTGCAC |
| KB303 | ATGTCGTTCGTGTTGATCGCAC |
| KB304 | GGTACCACCGGTTAGACCTGTGC |
| KB305 | CACCGGTAGTGCAGGCATC |
| KB306 | ATTGCCGCCAGCACCTCC |
| KB307 | TCGCCTGCCACAGCTTCTTC |
| KB308 | GTTACCTCGGTGGCTTGCAGTTC |
| KB309 | ATGCGCTTCCATCGCCAC |
| KB310 | CTGATCTCGGGCAGCTACAGC |
| KB311 | AGCGTGCTGGTGTTGTAGAGGC |
| KB312 | CGACTTCAACACGGGCAGCTC |
| KB313 | TGACGGTGACGGCGGTAC |
| KB314 | CTGGTGACGCCTGCTCCAC |
| KB315 | CACAACGCTTCACGGCACTC |
| KB316 | TGGCATTATCAGCACCGGAGC |
| KB317 | GTCGGTAACGCCGGGATC |
| KB318 | CCGTTACCGCCGTTACCGAC |
| KB319 | CCAGGGTTCCCGCACCAC |
| KB323 | CATCGCACCAGGTATCGAGGAAC |
| KB324 | GCTGCCAGTTTGTACACCTCGC |
| KB325 | CACCACCAACCAGCCGATC |
| KB326 | CCGTACTGTCACCCGGCTAGC |

**References with additional file 1**

1. Parish T, Stoker NG. Use of a flexible cassette method to generate a double unmarked Mycobacterium tuberculosis tlyA plcABC mutant by gene replacement. Microbiology. 2000;146:1969–75.

2. Festjens N, Vandewalle K, Houthuys E, Plets E, Vanderschaeghe D, Borgers K, et al. SapM mutation to improve the BCG vaccine: Genomic, transcriptomic and preclinical safety characterization. Vaccine. 2019. doi:10.1016/j.vaccine.2019.05.022.

3. Bedwell J, Kairo SK, Behr MA, Bygraves JA. Identification of substrains of BCG vaccine using multiplex PCR. Vaccine. 2001;19:2146–51.

4. Brosch R, Gordon SV, Garnier T, Eiglmeier K, Frigui W, Valenti P, et al. Mycobacterium bovis BCG Pasteur 1173P2, complete genome. NCBI Nucleotide Database. 2017. NC_008769.1.
